# Fanconi anemia and dyskeratosis congenita/telomere biology disorders: Two inherited bone marrow failure syndromes with genomic instability

**DOI:** 10.3389/fonc.2022.949435

**Published:** 2022-08-25

**Authors:** Moisés Ó. Fiesco-Roa, Benilde García-de Teresa, Paula Leal-Anaya, Renée van ‘t Hek, Talia Wegman-Ostrosky, Sara Frías, Alfredo Rodríguez

**Affiliations:** ^1^ Laboratorio de Citogenética, Instituto Nacional de Pediatría, Ciudad de México, Mexico; ^2^ Maestría y Doctorado en Ciencias Médicas, Universidad Nacional Autónoma de México (UNAM), Ciudad Universitaria, Ciudad de México, Mexico; ^3^ Departamento de Genética Humana, Instituto Nacional de Pediatría, Ciudad de México, Mexico; ^4^ Facultad de Medicina, Universidad Nacional Autoínoma de Meíxico (UNAM), Ciudad Universitaria, Ciudad de México, Mexico; ^5^ Subdirección de Investigación Básica, Instituto Nacional de Cancerología, Ciudad de México, Mexico; ^6^ Departamento de Medicina Genómica y Toxicología Ambiental, Instituto de Investigaciones Biomédicas, Universidad Nacional Autónoma de México (UNAM), Ciudad de México, Mexico; ^7^ Unidad de Genética de la Nutrición, Instituto Nacional de Pediatría, Ciudad de México, Mexico

**Keywords:** Fanconi anemia (FA)/BRCA pathway, dyskeratosis congenita (DC), telomere biology disorders, genomic instability, PHENOS, VACTERL-H, GINPOD, cancer risk

## Abstract

Inherited bone marrow failure syndromes (IBMFS) are a complex and heterogeneous group of genetic diseases. To date, at least 13 IBMFS have been characterized. Their pathophysiology is associated with germline pathogenic variants in genes that affect hematopoiesis. A couple of these diseases also have genomic instability, Fanconi anemia due to DNA damage repair deficiency and dyskeratosis congenita/telomere biology disorders as a result of an alteration in telomere maintenance. Patients can have extramedullary manifestations, including cancer and functional or structural physical abnormalities. Furthermore, the phenotypic spectrum varies from cryptic features to patients with significantly evident manifestations. These diseases require a high index of suspicion and should be considered in any patient with abnormal hematopoiesis, even if extramedullary manifestations are not evident. This review describes the disrupted cellular processes that lead to the affected maintenance of the genome structure, contrasting the dysmorphological and oncological phenotypes of Fanconi anemia and dyskeratosis congenita/telomere biology disorders. Through a dysmorphological analysis, we describe the phenotypic features that allow to make the differential diagnosis and the early identification of patients, even before the onset of hematological or oncological manifestations. From the oncological perspective, we analyzed the spectrum and risks of cancers in patients and carriers.

## 1 Introduction

The inherited bone marrow failure (BMF) syndromes (IBMFS) are a group of genetic and hereditary diseases characterized by common childhood onset exhaustion of the hematopoietic stem and progenitor cells (HSPCs) pool, a high frequency of extra-medullary phenotypes, and variable degree of cancer risk (1). BMF at any age should prompt a consideration of a IBMFS diagnosis (2). The two most frequent and probably best characterized syndromes of this group are Fanconi anemia (FA) that results from germline pathogenic variants (PVs) in genes of the FA/Breast Cancer Susceptibility (BRCA) DNA repair pathway and dyskeratosis congenita (DC) in which telomere maintenance genes are affected (3, 4). Recently, the designation telomere biology disorders (TBDs) have gained relevance for describing the spectrum of phenotypes associated with the telomere maintenance defects, including DC (3, 4). Although FA and DC/TBD are recognizable different syndromes, they may share a frequent initial clinical presentation consisting of primary refractory pancytopenia, megaloblastosis, cutaneous dyschromia, and growth retardation. Before cellular phenotype testing and molecular investigations were possible, early literature proposed that DC was a variant of FA (5), implying that historically FA and DC/TBD have been considered differential diagnoses to each other. Currently, the DC/TBD Diagnosis and Management Guidelines consider performing chromosome breakage analysis to rule out FA in the work-up for BMF evaluation.

In this review, we present a thorough analysis contrasting the phenotype of FA and DC/TBD from molecular and cytogenetic findings to dysmorphological and oncological aspects, making a one-to-one comparison between both conditions. We make special emphasis in contrasting their physical characteristics and highlighting the differences that could help in the differential diagnosis.

## 2 Pathophysiology

### 2.1 FA/BRCA DNA repair pathway

The *FANC* genes protein products collaborate in the so-called FA/BRCA pathway (6). This is a biochemical ensemble that maintains genome integrity by protecting the DNA replication forks during the S phase of the cell cycle and by promoting DNA repair (7). DNA replication is a timely process triggered by the activation of multiple origin replication sites in the lineal eukaryotic chromosomes, starting multiple replication forks that will coordinately replicate the DNA molecule (8). DNA replication is achieved by DNA polymerases that move along with the replication fork machinery, incorporating nucleotides into a nascent daughter DNA strand (9). The DNA replication process is not seamless, and addition of nucleotides by the replication fork machinery can be interrupted by diverse structures occurring in the template DNA molecule (10). These structures include damaged DNA bases, DNA–protein complexes, DNA–RNA hybrids (R-loops), and certain DNA structures inherent to the complementary nature of the DNA bases, such as G quadraplexes (10, 11). All these events have the capacity to dampen or slow down the progression of the replication forks and may retard the timely replication of the entire DNA molecule.

**Figure 1:**
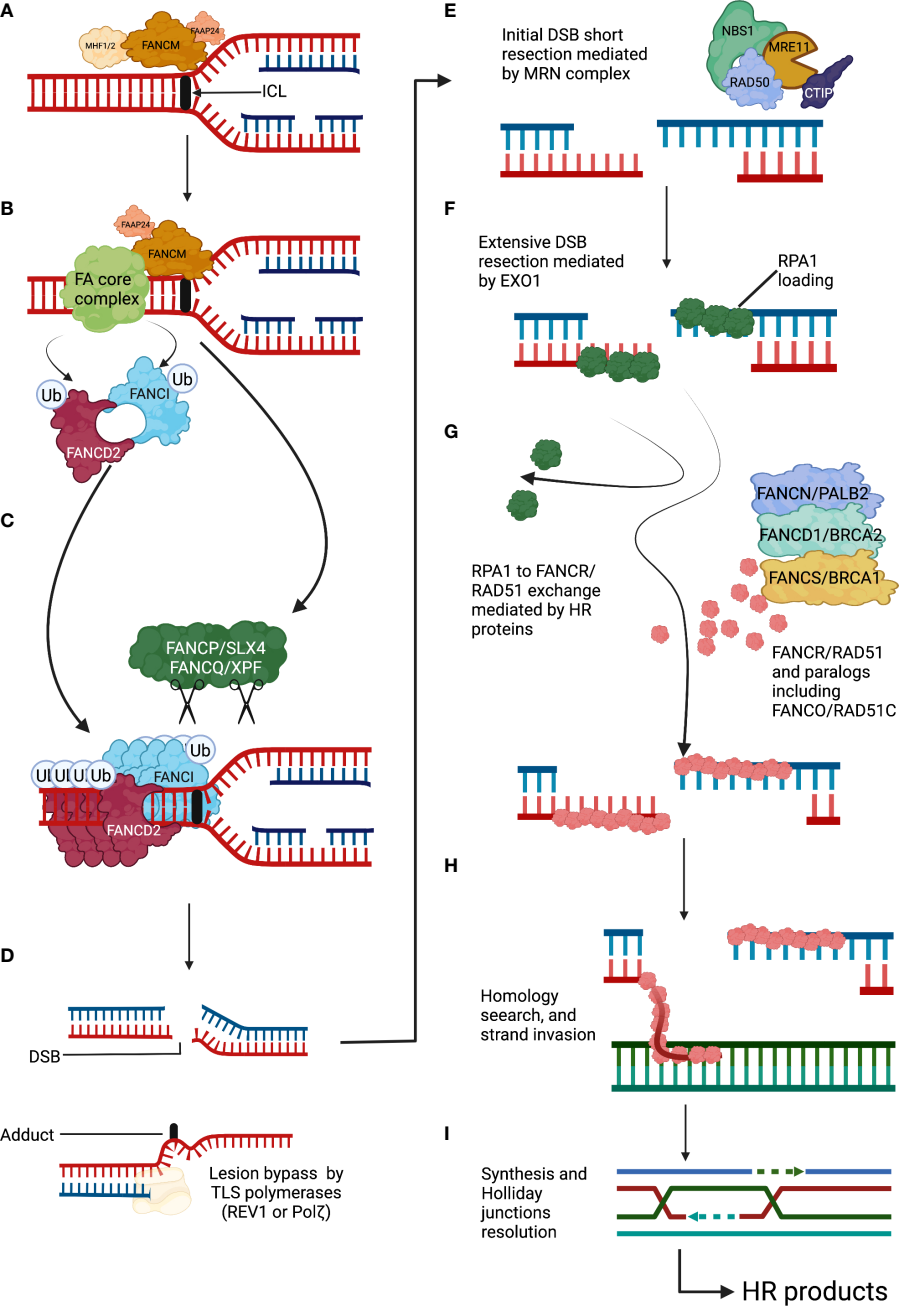
The Fanconi anemia pathway detects and repairs DNA interstrand cross-links. **(A)** An ICL covalently holds together the two complementary DNA strands. ICLs arrest the progression of the replication fork and are recognized by FANCM and its associated proteins. **(B)** The FANCM anchor complex recruits the FA core complex to the ICL site. The FA core complex in turn ubiquitylates the FANCD2-I complex. **(C)** Ubiquitylated FANCD2-I complex forms a filamentous array that clamps the stalled DNA replication fork and protects it from nucleolytic degradation. The FANCP/SLX4 is a DNA endonucleases toolbox that will unhook the ICL site by cleaving the surrounding DNA. **(D)** After ICLs unhooking two DNA repair intermediates appear, a DSB that will be repaired by the downstream FA/BRCA proteins using HR and a DNA adduct that will be processed with the help of TLS DNA polymerases. **(E)** The DSB will initially undergo a short resection mediated by exonuclease activity of the MRN complex. **(F)** Extensive DSB resection will be performed by the EXO1 endonuclease, and the produced DNA overhang will be covered and protected by RPA1 protein subunits. **(G)** The HR mechanism will start with the exchange of RPA1 by subunits of the recombinase RAD51. This exchange will be assisted by homologous recombination mediators including FANC/PALB2, FANCD1/BRCA2, and FANCS/BRCA1. **(H)** RAD51-coated DNA overhangs will function as recombinase filaments that will search for sequence homology. **(I)** Strand invasion will be followed by sequence detection, copy and synthesis of the sequence of interest, Holliday junctions resolution, and ligation of the phosphate DNA backbone. DSBs, double-strand breaks; FA, Fanconi anemia; HR, homologous recombination; ICLs, interstrand cross-links; TLS, translesion synthesis.

The better known function of the FA/BRCA pathway is the removal of interstrand cross-links (ICLs) in the DNA molecule (7) (**Figure 1**). ICLs are structures covalently binding both DNA strands that interfere with DNA replication and genetic transcription by impeding the separation of the two DNA strands (10). ICLs can have an endogenous origin: Normal metabolic transactions of the cell generate by-products, which include aldehydes, reactive oxygen species, and nitrous acid, with the capacity to react with the DNA molecule (12, 13). They can also have an exogenous origin, ICLs inducing drugs are a cornerstone of cancer chemotherapy, and it is precisely the replicative damage that they generate what leads to apoptosis activation in cancer cells. Frequently used agents include platinums and nitrogen mustards (10).

The FA/BRCA pathway comprises a series of coordinated actions that allow (1) the recognition of the ICLs (2), the removal of the ICLs with the generation of a double-strand break (DSBs) as a DNA repair intermediate; and (3) the repair of the DSB by homologous recombination (HR) (6, 7) (**Figure 1**).

ICLs are recognized during S phase, mainly by the UHRF1 protein which, together with an anchor complex composed of Fanconi Anemia Complementation Group M (FANCM) and FAAP24, ensure the recruitment of the FA core complex and the FANCD2-I heterodimer to the appropriate location in the chromatin (14). The positioning of these complexes constitutes the upstream portion of the FA/BRCA pathway (15–18). The FA core complex is a large protein assembly with E3 ubiquitin ligase enzymatic activity, integrated of at least 10 proteins (FANCA, FANCB, FANCC, FANCE, FANCF, FANCG, FANCL, FAAP100, and FAAP20) (17). The FA core complex recruits the E2 conjugating enzyme UBE2T/FANCT to monoubiquitylate the FANCD2-I complex (Ub-FANCD2I) (19, 20). Ub-FANCD2I is being long recognized as essential for the recruitment of proteins involved in the DNA repair downstream activities of the FA/BRCA pathway (21), as FANCD2-I forms nuclear DNA repair foci co-localize with DNA repair factors (22). However, recent structural studies have shown that the recruitment of additional DNA repair factors to the sites of damage occurs independently of Ub-FANCD2-I and, indeed, Ub-FANCD2-I forms a nucleoprotein array that clamps around stalled replication forks. This Ub-FANCD2-I clamp may, instead of recruiting DNA repair factors, protect the DNA replication fork by preventing the activity of DNA nucleases, such as MRE11 and DNA2, that would resect and destabilize the DNA replication fork (23–25). Importantly, cells from patients with FA with PVs in components of the FA core complex do not have the capacity to ubiquitylate the FANCD2-I complex and cannot make these critical foci that maintain genome stability (17).

FANCP/SLX4 is a scaffolding protein that allows the engagement of multiple DNA endonucleases: MUS81, SLX1, and FANCQ/XPF/ERCC4 (26, 27). FANCP/SLX4 is thought to be recruited by Ub-FANCD2-I, but recent reports have also shown that FANCP/SLX4 can directly recognize stalled replication forks and can be recruited to the site of damage independently from Ub-FANCD2 (28, 29). The endonucleases coordinated by FANCP/SLX4 would cleave the phospho-backbone of the double stranded DNA, cut the DNA strand contiguous to the ICLs, and generate a DNA adduct and a DSB (30). These two secondary DNA lesions need to be further funneled into appropriate downstream pathways for repair. REV1 and REV3 are low-processivity translesion synthesis DNA polymerases with the capacity to bypass the DNA adduct generated after ICL incision and ensure the continuity of the DNA replication process (31), whereas HR, a high-fidelity DSBs repair pathway, takes charge of the DSB generated after ICL incision (7). Switching off the FA/BRCA pathway is also relevant, and this happens when the USP1–UAF1 complex deubiquitylates the FANCD2-I complex, adding an additional layer of regulation to the FA/BRCA pathway (17).

The FA/BRCA pathway blocks the activity of alternative low-fidelity DNA repair pathways, including the non-homologous end-joining (NHEJ) pathway, and promotes the DSB repair by HR (32). This ensures fidelity in the repair of the DSB generated after ICL cleavage. In this high-fidelity pathway, the ends of the DSB are processed by the DNA exonucleases CtIP and the MRN (MRE11-RAD50-NBS1) complex, initially performing a short resection, followed by an extended resection that is mediated by EXO1 (33). This generates a 3′ single-stranded DNA (ssDNA) overhang that is coated by replication protein A1 (RPA1), a protein that prevents formation of secondary structures in the ssDNA due to auto-complementarity (34). Eventually, RPA1 is evicted from the ssDNA and is substituted by FANCR/RAD51, forming a recombination filament that performs homology search in the sister chromatid (35). The recombination filament invades the homologous sequence aided by the FANCD1/BRCA2, FANCN/PALB2, FANCO/RAD51C, FANCJ/BRIP1/BACH1, and FANCU/XRCC1 proteins, when it detects enough homology, it copies the sequence of interest aided by DNA polymerases (7, 21). Of note, cells from patients with FA who have PVs in downstream components of the FA/BRCA pathway can ubiquitylate FANCD2-I foci in the presence of ICLs (36). These cells can detect the ICLs and perform the initial ICLs unhooking; however, they are not able to perform the accurate repair of DSB by HR. Patients with PVs in the downstream *FANC* genes are even more rare than patients with PVs in the upstream FA/BRCA pathway or FANCD2-I complex (37–39). This may probably be due to early lethality since lack of HR proteins will affect the repair not only of DSB generated during ICL processing but also the repair of direct DSB, thus affecting the tolerability to more DNA damaging agents and processes.

### 2.2 The telomere maintenance system

**Figure 2:**
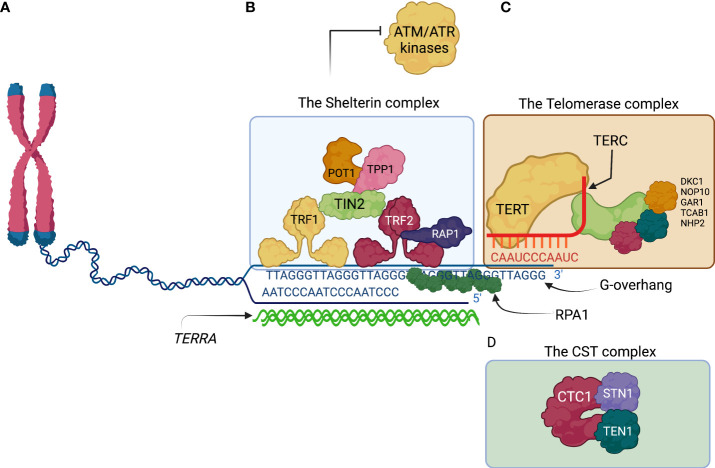
The ends of the human lineal chromosomes are protected by telomere associated proteins. **(A)** The lineal nature of the human chromosomes possesses a challenge for integrity maintenance of the human genome. The telomeres at the end of the chromosomes consist of repetitive sequences TTAGGG and their complementary sequence AATCCC. A G-overhang composed by the TTAGGG sequence is the result of incomplete DNA fill-in activity by DNA polymerases at every round of DNA duplication. The G-overhang is bound by RPA1 subunits conferring protection from nucleolytic degradation. **(B)** Shelterin is telomere protecting complex that is composed by six proteins. TRF1 and TRF2 directly bind the telomeric DNA sequences and recruit other members of the Shelterin complex. Shelterin promotes the t-loop lariat conformation of the G-overhang and inhibits activation of the checkpoint kinases, ATM by TRF2, and ATR by POT1. **(C)** The telomerase complex is a ribonucleoprotein whose main components are TERT and TERC proteins. TERT synthetizes telomeric DNA using TERT as a template and extends the TTAGGG overhang. **(D)** The CST complex is composed by the CTC1, STN1, and TEN1. CST complex recruits Pol-a-primase to help fill in the recently extended TTAGGG with the complementary AATCCC. CST complex also inhibits telomerase activity, preventing excessive telomere extension. ATM, ataxia telangiectasia mutated; ATR, ATM and Rad3 related; CTC1, conserved telomere protection component 1; POT1, protection of telomeres 1; RPA1, Replication Protein A1; STN1, suppressor of cdc thirteen 1; TEN1, telomeric pathway with STN1; TERC, telomerase RNA template component; TERRA, telomeric repeat-containing RNA; TERT, telomerase reverse transcriptase; TRF1, telomeric repeat binding factor 1; TRF2, telomeric repeat binding factor 2.

The linear chromosomes found in eukaryotic cells need protection of their ends from nuclease degradation and require special structures and mechanisms for this task. The telomere is a complex structure located at the ends of the linear chromosomes that accomplishes this protective role. Telomeres are composed of repetitive DNA sequences coated by specialized proteins, ribonucleoprotein complexes, and long-noncoding RNA (40–42) (**Figure 2**).

The telomeric DNA sequence is highly conserved among vertebrates; it is composed of the repetitive hexameric sequence TTAGGG (43). In human chromosomes, it can extend for up to 5 kb (44), whereas, in mice, it may go up to 100 kb (45). The architecture of the telomeric DNA is special; it is composed of a region of double-stranded DNA that is several kilobases in length, where the TTAGGG hexamer and its complementary sequence AATCCC are repeated. Eventually, the AATCCC rich strand is discontinued and only the single-stranded 3′ tail known as the G-overhang continues (enriched in the TTAGGG sequence) (41). The G-overhang invades the preceding double-stranded region generating a lariat-like structure known as the telomere loop or *t-loop* (42). Importantly, the telomeric length of the chromosomes is progressively shortened with every cell division and serves as a “molecular clock” of the proliferative life span of primary cells (46). Telomeric lengths among chromosomes of the same cell, tissue, and organism can be highly heterogeneous (47). Of upmost relevance, critically short telomeres are known to trigger the entrance of the cell to senescence, also known as the Hayflick limit of proliferative life span, a mechanism that controls the tissues life span and regeneration (46).

At least three different protein complexes with different and specific functions have been associated with the telomeres (**Figure 2**). First, the shelterin complex, a six-subunit protein complex that binds directly to the telomeric repeats and protects chromosome ends by inhibiting the activation of the DNA damage response (DDR) (explained below) (48). The human shelterin complex includes six proteins: telomere repeat binding factor 1 (TRF1), TRF2, repressor/activator protein 1 (RAP1), TRF1-interacting nuclear protein 2 (TIN2), TIN2-interacting protein 1 (TPP1), and protection of telomeres 1 (POT1) (40, 41, 48).

The second telomere-associated complex elongates the TTAGGG telomeric sequences through reverse transcriptase activity. This ribonucleoprotein complex is a telomerase that synthesizes new telomere sequences onto chromosome ends (49). The telomerase holoenzyme has two components: Telomerase Reverse Transcriptase (TERT), the core telomerase protein, which contains the telomerase reverse transcriptase domain; and Telomerase RNA Component (TERC), the RNA component, which provides the template for telomeric sequence synthesis (49). The human telomerase is assembled in the Cajal bodies (50).

The telomerase complex relies on other proteins for its assembly and forms a ribonucleoprotein complex with several accessory proteins, among which dyskerin is the best characterized among mammalian telomerase complexes and the most frequently affected gene (*DKC1*) in patients with DC/TBD (49, 51). Three other smaller accessory proteins accompany dyskerin, namely, NHP2, NOP10, and GAR1 (49, 52). Dyskerin associates with TERC, the RNA component of the complex, through its binding to an H/ACA box structural motif within TERC, which is essential for TERC stability and telomerase function (49, 53). Telomerase uses its intrinsic RNA template to synthesize telomeric DNA repeats and has the capacity to add ~60 nucleotides per telomere each cell cycle. The telomerase complex is recruited by the shelterin complex (41). Importantly, telomerase is more abundant in germline cells and is absent or less abundant in somatic cells (41, 54).

A third complex is the CST complex, composed of three proteins: CTC1, STN1, and TEN1. The CST complex aids in terminating the telomeric extension performed by the telomerase complex. CST complex binds to the newly extended single-stranded DNA and facilitates the recruitment of DNA polymerase alpha-primase (Pol α-primase) (41, 55). Pol α-primase fills in the C-strand (AATCCC), complementary to the recently extended TTAGGG overhang, thus helping convert part of the recently extended tail into double stranded DNA, leaving the overhang length to ~50–300 nucleotides (55).

A less-well characterized component of the telomeric complex is the long non-coding telomeric repeat-containing RNA (TERRA), which is transcribed by the RNA polymerase II from intrachromosomal telomeric repeats. Although TERRA function is not fully understood, it may work as a scaffold for telomeric proteins (mentioned below) (56).

### 2.3 Defects in both the FA/BRCA pathway and the telomere maintenance system exacerbate the cellular DNA damage response

**Figure 3:**
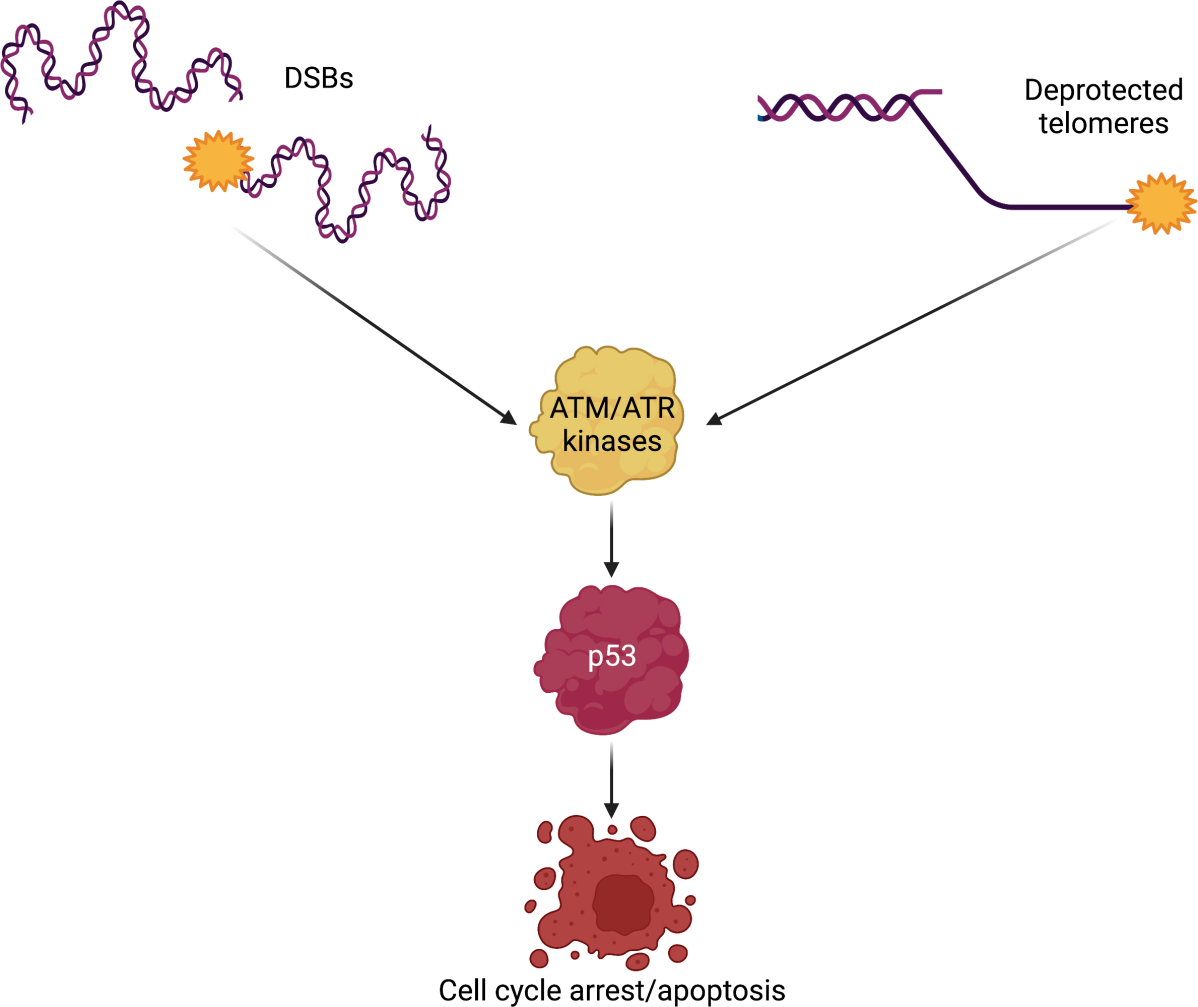
DSBs and deprotected telomeres trigger similar DNA damage signaling. The members of the PIKK family of kinases are activated by DNA damage. Both DSBs, generated either by exogenous or endogenous sources, and deprotected telomeres are recognized as DNA damage that must be repaired. Excessive DNA damage leads to activation of p53, the guardian of the genome. p53 is a transcription factor with the capacity to control the activation of cell cycle arrest genes when DNA damage is repairable; however, unsurmountable amounts of DNA damage will lead the cell into apoptosis, also in a p53-dependent manner. p53-mediated apoptosis in response to DNA damage may be a common mechanism leading to HSPCs demise in Fanconi anemia and DC/TBD. DSBs, double-strand breaks; DC/TBD, dyskeratosis congenita/Telomere biology disorders; HSPCs, hematopoietic stem and progenitor cells; PIKK, phosphatidylinositol-3 kinase-related kinases.

The FA/BRCA pathway and the telomere maintenance system promote genome stability (6, 48). Importantly, both are linked, in opposing ways, to the DDR that monitors the integrity of the genome. On the one hand, the presence of DNA damage promotes activation of the cell cycle checkpoints, halts cell cycle progression, and allows the FA/BRCA pathway to perform repairing activities (7). On the other hand, the relationship between the telomere associated proteins and the DDR is the opposite; the components of the shelterin complex inhibit the activation of the DDR; this prevents the telomeric ends to be recognized as unrepaired DSB and joined with other DSB (57) (**Figure 3**).

In this context, the main DDR orchestrators are the members of the PIKK family of kinases, namely, ATM (ataxia telangiectasia mutated), ATR (ataxia telangiectasia and Rad3-related), and DNA-PKCs. ATM is known to signal DSB, whereas ATR recognizes stalled replication forks and RPA1-coated single-stranded DNA (58, 59). The best known ATM target is the checkpoint kinase 2 (CHK2) (60), whereas for ATR, it is CHK1 (61). The ATM–CHK2 axis, as well as the ATR–CHK1 axis, phosphorylates p53, a master transcription factor that activates the expression of proteins that block cell cycle progression (62). In this sense, the presence of DNA damage, in the form of ICLs and DSBs, promotes p53 activity to block cell cycle progression and allowing DNA repair to occur. In contrast, if the DNA damage is excessive, p53 activity will promote cell death by apoptosis (63) (**Figure 3**).

ATM establishes an activation loop with the MRN complex (64). The MRN complex has an endonuclease activity that resects DSB and generates ssDNA that will be coated by RPA1 subunits (65); this will subsequently activate ATR and reinforce the checkpoint signaling (59). In the context of the FA/BRCA pathway, ATR will be involved in the initial recognition of ICLs, will phosphorylate FANCM (66, 67), as well as Fanconi Anemia Complementation Group I (FANCI) (23, 68) for promoting the activation of the FA pathway.

At the telomeres, the story is the opposite, the illegitimate repair of telomeric ends is actively inhibited (48). Unleashing the DNA repair activity at the telomeres can have catastrophic consequences, including the formation of end-to-end chromosome fusions, resulting in dicentric chromosomes that will conflict the chromosome segregation process during mitosis (69–71). This can lead to breakage–fusion–bridge (BFB) cycles and chromothripsis, a single event of thousands of genomic breakages followed by chromosomal rearrangements clustered in a confined genomic region in one or a few chromosomes (72). These alterations generate great amounts of structural chromosomal damage (explained in detail in the next section). This repressive activity lies on the components of the shelterin complex that actively inhibit the PIKK kinases. More specifically, TRF2 inhibits ATM and POT1 inhibits ATR (73).

In the absence of TRF2, ATM phosphorylates its targets CHK2 and p53. The later activates the recruitment of DNA damage repair proteins at the unprotected telomeres forming telomere dysfunction-induced foci (74). These foci are characterized by the presence of yH2AX and recruitment of 53BP1 (p53-binding protein 1), a well-known NHEJ protein (74). TRF2 also blocks the dimerization of the Ku complex, thereby preventing the activation of NHEJ (75, 76). Therefore, TRF2 is a multipurpose protein that inhibits the checkpoint activation and NHEJ at the chromosome ends, whereas POT1 inhibits alt-EJ of DSB (77).

Two mechanisms have been proposed to explain how TRF2 inhibits ATM. The first involves the *t-loop* formation; this lariat-like structure is stimulated by TRF2 and helps hiding the telomere ends from being detected by MRN (78, 79). The second prevents further ATM activation and avoids the DNA ends to be processed by the nucleolytic activity of MRN. TRF2 might also inhibit ATM directly by inhibiting its kinase activity (80).

POT1 is an ssDNA-binding protein specially located at the telomeric ends (81). POT1 binds directly to the telomeric ssDNA and, with the help of the heterogeneous nuclear ribonucleoprotein A1 and TERRA, displaces RPA, avoiding its recruitment, preventing the accumulation of RPA-coated ssDNA (**Figure 2**), the critical substrate needed for ATR activation (81). POT1 depletion leads also to the formation of telomere dysfunction-induced foci in an ATR-dependent manner. ATR can also be inhibited by TRF1; this happens during telomere replication with the help of TPP1 and POT1 (82).

When the FA pathway is nonfunctional or the telomere maintenance system fails, there will be checkpoint activation and the subsequent activation of p53. Of note, activation of p53 has been observed in HSPCs from patients with FA (83) and human embryonic stem cells with PVs in genes relevant for DC/TBD (84). Altogether, this suggests that p53 is the main executor of the cell death program in both diseases, leading to BMF by depletion of the HSPCs pool. Abrogation of p53 rescues the proliferation capacity of FA cells; however, losing p53 promotes the division of cells with great amounts of DNA damage and potentially to malignancy (83, 84). Recently, overexpression of *MYC*, a proto-oncogen, was observed in HSPCs from patients with FA, presumably as a counterbalancing mechanism against the p53-mediated cell death. *MYC*, however, produces great levels of replication stress and DNA damage that eventually might reinforce p53 activation, in what seems to be a vicious cycle (85). *MYC* activation has not been studied in DC/TBD, but *MYC* may also be a force allowing the survival of cells deficient in telomere protection.

### 2.4 Chromosomal instability in FA and DC/TBD

#### 2.4.1. Fanconi anemia

The FA/BRCA pathway is essential for the repair of ICLs (4, 6). Having a non-functional FA/BRCA pathway has several consequences; one of them is that ICL-derived DSB will not be repaired error free by HR (4). For this reason, FA cells are highly sensitive to endogenous and exogenous agents that generate ICLs (7, 75). Some examples are as follows: cisplatin and mitomycin C (MMC), drugs that are used in the treatment of cancer, and also diepoxybutane (DEB) (12, 86).

FA cells, therefore, rely on low-fidelity alternative DNA repair pathways, including the canonical NHEJ (4, 87) and potentially the alternative microhomology-mediated end-joining pathway (MMEJ). Both pathways are error prone, and their excessive use increases the frequency of chromosomal abnormalities, mainly of the structural type.

The processing and repair of DSBs are influenced by several factors, including the cell cycle phase and the number of DSBs simultaneously present in the same cell (88, 89).

**Figure 4:**
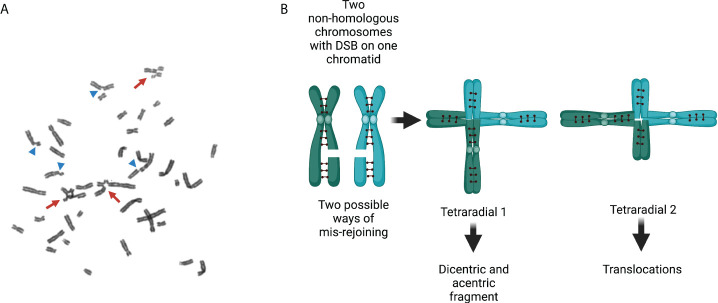
Chromosomal aberrations in Fanconi anemia. **(A)** Exposure of FA cells to ICL-inducing agents increases the number of structural chromosome aberrations and the number of aberrations per cell. Arrows show radial figures, and arrowheads show chromatid breaks; both are characteristic chromosomal aberrations found in FA cells. **(B)** Representation of two replicated non-homologous chromosomes with their sister chromatids linked by cohesins and each with a DSB in only one chromatid. Two types of radial figures may arise when the DSBs in two different replicated chromosomes are mis-rejoined. Tetraradial 1 results from the end joining of two chromosome proximal segments. Mitotic segregation of tetraradial 1 produces daughter cells with a dicentric chromosome and an acentric fragment (originated by the ligation of the two distal segments). Tetraradial 2 results from the joining of one proximal and one distal segment of two different chromosomes. Mitotic segregation of tetraradial 2 results in daughter cells with translocations. DSBs, double-strand breaks; FA, Fanconi anemia; ICLs, interstrand cross-links.

In an FA cell, the HR pathway is deficient and the number of DSBs is high. Under these conditions, FA cells recur to error prone end-joining repair mechanisms that operate throughout the entire cell cycle. The emergency posed by the presence of several DSBs may be solved with the activity of alternative end-joining pathways, which intrinsically are template-independent and need a minimal homology for joining free DNA ends (**Table 1**) (90, 91). However, the potentially indiscriminate ligation of DNA that ends from different chromosomes may lead to gross chromosomal abnormalities (4). When metaphase spreads from patients with FA are analyzed, chromatid breaks can be observed, which is the evidence of unrepaired DSBs, as well as radial figures and other types of structural chromosomal aberrations, resulting from the erroneous ligations of DSBs coming generally from chromatids of non-homologous chromosomes (**Figure 4**) (92–94).

**Table 1:** Characteristics of the major double-strand breaks repair pathways.

	Speed	Template dependence	Homology usage	End resection	Cell cycle phase	Accuracy
**Homologous Recombination**	Slow	Dependent	>100 base pairs	Yes	S, G2	Highly accurate
**Microhomology Mediated End-Joining**	Fast	Independent	2-20 base pairs	Yes	G1, S, and G2	Error prone
**Non homologous End-Joining**	Fast	Independent	0-4 base pairs	No	G1, S, and G2	Error prone

References (90, 91).

Radial figures originate in S/G2 phases, when two chromatids become available from two replicated chromosomes. Unrepaired DSBs lead to chromatid breaks and, when rejoined by the error-prone DNA repair pathways, radial figures are produced after the ligation of chromatids from different chromosomes. The abnormal processing and repair of the ICLs might generate multiple types of complex structural chromosomal abnormalities, including translocations, deletions, or dicentric chromosomes. Abnormal segregation of dicentric chromosomes during mitosis may also be a source of complex rearrangements.

**Figure 5:**
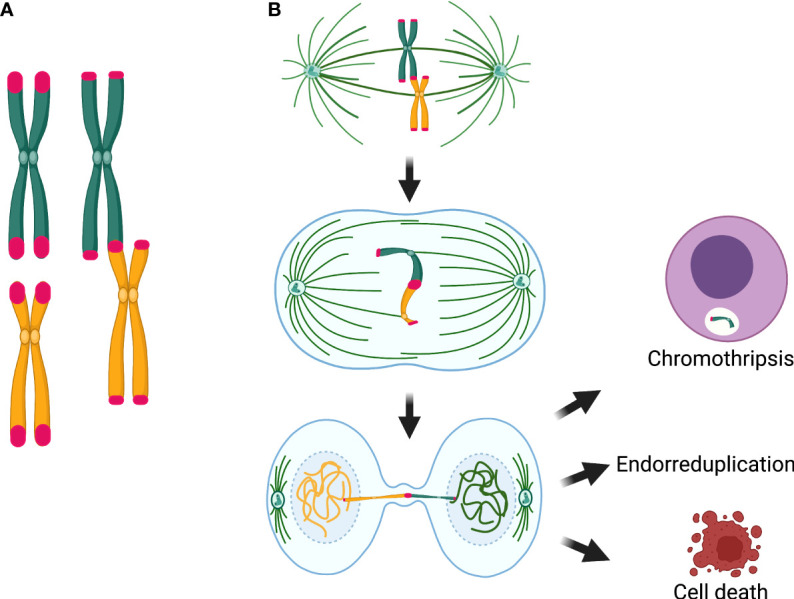
Chromosomal consequences of telomere attrition. **(A)** Telomere attrition produces telomere fusions that originate dicentric chromosomes. **(B)** Segregation of the dicentric when the two centromeres are pulled by opposite poles of the mitotic spindle is the main source of chromosome and cell abnormalities, because the dicentric may induce a breakage–fusion–bridge cycle. If the chromatin of the dicentric bridge is enzymatically fragmented or if the dicentric breaks and a micronucleus is formed, gross structural rearrangements, such as chromothripsis, may arise in the daughter cells. Alternatively, when chromatin bridges are not resolved, interruption of mitosis and cytokinesis, may lead to polyploid cells and endorreduplicated chromosomes in the next mitosis. Until now, chromothripsis has not been detected in cells from patients with DC/TBD; however, anaphase bridges, fragmentation of a single chromosome, which is the precedent of chromothripsis, endoreduplications, and polyploidy have been found in DC cells.

Translocations, deletions, and duplications may arise when the two centromeres of a dicentric chromosome are pulled to different mitotic poles during anaphase (**Figure 5**). These structural aberrations are frequently accompanied by numerical abnormalities, such as aneuploidies and endoreduplications. Endoreduplication occurs when a cell enters a new round of DNA replication in the absence of cytokinesis, causing a numerical anomaly leading to the appearance of “double” chromosomes in metaphase spreads when the cells with the endorreduplicated material reach mitosis (95).

The presence of chromosomal instability (CI) is used for FA diagnosis by comparing the frequency of chromosomal abnormalities in the subject sample after ICLs induced damage, to those produced in a negative control (a healthy donor) and, ideally, a positive control (a FA cell line) (92, 94, 96). The presence of radial figures and an ~10-fold increase in the frequency of chromosomal abnormalities indicates a positive diagnosis for FA (92, 93). Importantly, the genomic instability that characterizes FA can generate revertant mutants that recover the DNA repair capacity. Up to 30% of patients with FA may be hematologic mosaics based on normalization of their blood counts. Nevertheless, two studies that intentionally looked for somatic reversion found that only 15% of patients developed mosaicism (97, 98). A recent review of FA mosaic publications with emphasis on blood count normalization alerted of a possible publication bias and described that some degree of mosaicism is present in around 15% of FA cases, but the incidence of PV reversion occurs in fewer than 5% (99). Therefore, when the frequency of chromosome aberrations and the number of aberrant cells shows intermediate values compared with a positive control, a mosaic must be suspected. In these cases, the FA diagnosis must be confirmed in skin fibroblasts, for which revertant mosaicism has not been described and should retain the MMC/DEB sensitivity (99).

Chromosomal damage can be lethal, and cell death may be activated by accumulation of gross chromosomal alterations or by mitosis blockage; however, non-lethal DNA damage may create a vicious cycle of non-clonal chromosomal aberrations and CI, rendering a heterogeneous cell population from which natural selection might pick a successful clone that can evolve into cancer (4).

#### 2.4.2 DC/Telomere biology disorders

Telomeres are structures that protect the chromosome ends from erosion and illegitimate fusion with other chromosomes (91). Telomerase and telomere-stabilizing proteins maintain the normal length and function of telomeres in stem cells of some tissues, such as germ cells, HSPCs, activated T-cells, monocytes, skin, and intestinal lining; therefore, the lifelong maintenance and correct function of these cells depend on the telomerase activity (100).

DC/TBD patients have an excessive shortening of telomeric repeats, below the first percentile for age, and the telomeres become uncapped (101). Cells detect this as a DSB and trigger the DDR (102, 103). Erroneous rejoining of DSBs may result in telomere-telomere fusions between chromosomes, provoking structural chromosomal aberrations, such as dicentric or multicentric chromosomes.

During mitosis, a dicentric chromosome may be captured by microtubules from opposite poles of the mitotic spindle, causing anaphase/telophase bridges; this affects the proper completion of mitosis and promotes tetraploidization and aneuploidy. Dicentric bridges may persist through mitosis and cytokinesis, inducing the chromosome BFB cycles that directly affect chromosomal segregation. It has been proposed that resolution of the chromatin bridges implies the formation of ssDNA segments and formation of micronuclei containing whole chromosomes or chromosome fragments. These anomalies might lead to chromothripsis, yet the latter has only been observed *in vitro* (72).

Telomere shortening in DC/TBD has been widely documented (3, 104). However, the formation of dicentrics and their consequent CI remain controversial due to contradictory results. On several occasions, the presence of structural and numerical alterations, both spontaneous and induced, has been intentionally sought (98, 105–107). Most of these reports have been negative when the spontaneous chromosomal aberrations and the MMC/DEB-induced DNA damage have been interrogated (98, 105–107). However, other studies report positive results, with elevated percentage of spontaneous structural alterations, including chromatid and chromosomal breaks (108, 109), as well as DNA damage induced by X-radiation, 4-nitro-quinoline-1-oxide (4NQO), and bleomycin (107, 110, 111). Still, Dokal et al. did not find hypersensitivity to DEB, MMC, 4NQO, bleomycin, or gamma radiation in fibroblasts from patients with DC/TBD (112).

The different findings on the cytogenetics of DC/TBD may be related to the type of cells studied. For example, a single report showed no spontaneous chromosomal aberrations in peripheral blood lymphocytes, but those anomalies were found in skin fibroblasts from the same patient (102). The site of the biopsy from which the cells are cultured can also influence the cytogenetic findings, since they can represent tissues in a precancerous state, for example, when the aberrations are studied from bone marrow that may be in the way of a neoplastic process or fibroblasts from cutaneous lesions (102, 103). The age of the patients has also been proposed as a source of variation, since older members of the same family display chromosomal aberration in skin fibroblasts, whereas the younger ones did not (112). Of note, dicentric chromosomes, expected in DC/TBD due to increased telomeres stickiness, could not appear spontaneously.

Interestingly, some cytogenetic studies have found non-classical chromosome abnormalities, such as pulverization (fragmentation of the entire chromatin) and fragmentation of individual chromosomes, whereas the rest of the chromosomes in the same metaphase spread appear normal (109, 113). Other genomic events found in DC/TBD cells include micronuclei, lagging chromosomes, and anaphase bridges (113), as well as endoreduplications and polyploidy (114). These types of cytogenetic findings may be important in the light of recent discoveries, according to which the BFB cycle can cause cell death (as pulverized mitosis) or very irregular mitosis that could survive if cytokinesis is interrupted (endoreduplication or polyploidy). BFB can also produce micronuclei, which can contain lagging chromosomes or fragments resulting from the breakage of dicentric chromosomes; the latter could finally cause fragmentation of a single chromosome, or the chromatin bridge may be enzymatically fragmented (TREX1-mediated) and result in chromothripsis (**Figure 5**) (40, 72).

All these data indicate that even though FA and DC/TBD share mechanisms that result in genomic instability and ultimately BMF, the cytogenetic features are evidently different. In FA, the presence of CI, both spontaneously and induced by MMC and DEB, is observed in practically all the cell types that have been studied from patients with FA. This characteristic is the hallmark that allows the cytogenetic diagnosis. In addition, CI is expected as a consequence of failure of the FA/BRCA pathway. In contrast, in DC/TBD, the presence of CI has not been definitively documented, even though telomeric attrition is well recognized and important in the diagnosis of DC/TBD. Finding only sporadically dicentric in cells from patients with DC/TBD is a fact that does not have a convincing explanation. It is possible that the difficult handling of dicentrics during cell division generates immediate cell death and, in cases in which the cell survives, the consequences at the chromosomal level could possibly be non-classical chromosomal abnormalities and chromothripsis, which conventional cytogenetic methods cannot detect. Molecular methodologies that can study the whole genome, such as whole genome sequencing and M-FISH, could help in the evaluation of chromosome instability in DC/TBD.

## 3 Dysmorphological and oncological phenotype of patients with FA and DC/TBD

**Figure 6:**
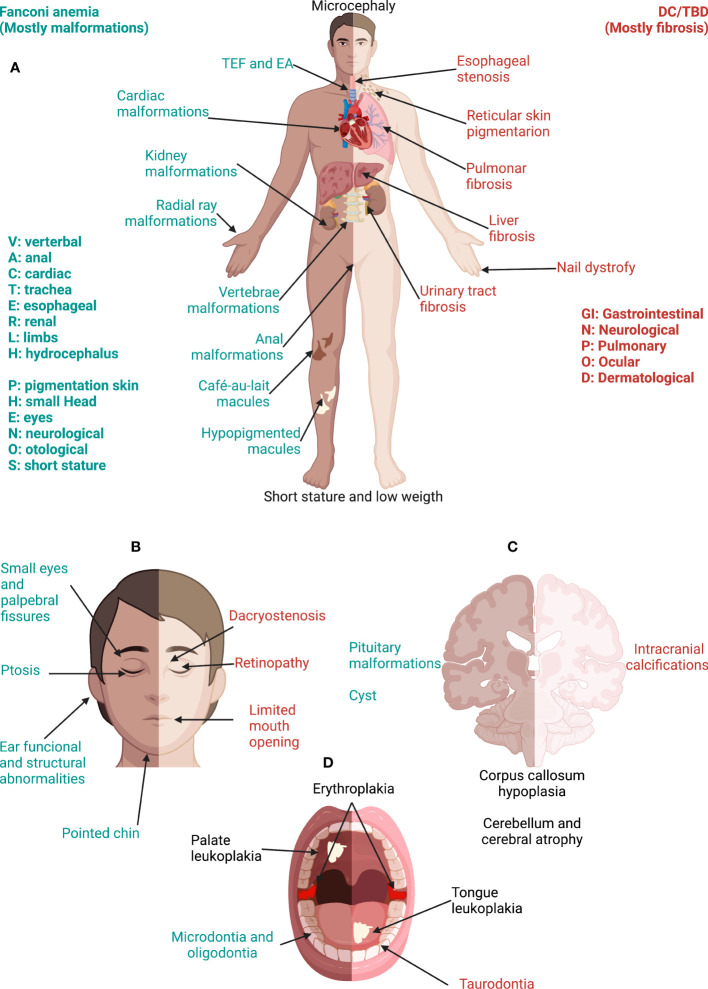
Phenotype in Fanconi anemia consists mainly in malformations whereas, in DC/TBD, it has a fibrotic nature. **(A)** The physical phenotype in FA is synthesized in the VACTERL-H (Vertebral, Anal, Cardiac, Tracheo-Esophageal, Renal, Limbs, Hydrocephalus) and PHENOS (Pigmentation of the skin, small Head, Eyes, Neurological, Otological, Short stature) acronyms. In DC/TBD, the physical phenotype can be informed by the GINPOD (GastroIntestinal [including liver and esophageal], Neurological, Pulmonary, Ocular, and Dermatological [including skin, nails, and leukoplakia]) acronym. GINPOD acronym includes the anomalies described in at least 20% of patients with DC/TBD. **(B)** Patients with FA have facial dysmorphism that integrate a recognizable FA facies: It includes small eyes and palpebral fissures, ptosis, and a pointed chin and can be complemented by ear abnormalities. In DC/TBD, the more striking facial findings are dacryostenosis and limited mouth opening. **(C)** At the nervous system level, patients with FA can have pituitary malformations and posterior fossa cysts, whereas patients with the HHS and RS, severe subtypes of DC/TBD, have calcifications. Both disorders may have corpus callosum abnormalities as well as cerebellum and cerebral atrophy. **(D)** Erythroplakia and leukoplakia are the more frequent oral findings in both FA and DC/TBD; tongue leukoplakia is more frequent in DC/TBD, whereas palate leukoplakia is more frequently found in FA. Dental phenotype is contrasting as FA has microdontia and oligodontia and DC/TBD has taurodontia. Features exclusive to FA are in green, those exclusive to DC/TBD are in red, and those shared by both disorders are in black. DC/TBD, dyskeratosis congenita/Telomere biology disorders; EA, esophageal atresia; FA, Fanconi anemia; HHS, Hoyeraal-Hreidarsson; RS, Revesz syndromes; TEF, Tracheoesophageal fistula.

The unifying characteristic of patients with DC/TBD is that they have short telomeres, yet the phenotypic presentation can be particularly broad as illustrated in **Figure 6** (3, 103). DC is the archetypal TBD. It was initially characterized by the so-called DC triad that consists of reticular skin pigmentation, nail dystrophy, and oral leukoplakia (115). Although, these features are not universal nor do they exist concomitantly in all patients with DC/TBD, but they are individually found in more than 80% of patients (116). The presence of the three classic triad components has been reported in 46% of patients with DC/TBD (115). Other consistent but less frequent physical abnormalities are pulmonary and liver fibrosis (116–118). On the severe side of clinical presentation, the DC/TBD subtypes known as Hoyeraal-Hreidarsson (HHS) and Revesz syndromes (RS) have an early presentation of BMF accompanied by neurological impairment. Specifically, HHS is associated with neurological and immunological abnormalities as well as intrauterine growth restriction (IUGR) (119–121), whereas RS includes retinopathy, neurological anomalies, and IUGR (3, 122).

DC/TBD have important genetic heterogeneity; at least 17 germline PVs in telomere biology genes have been associated with DC/TBD phenotype (**Table 2**) (3, 7, 118, 123–130). Autosomal dominant, autosomal recessive, and X-linked inheritance patterns have been described (3, 118) (**Table 2**).

**Table 2:** Genes, inheritance patterns, pathophysiology, and diagnostic strategies.

	Fanconi Anemia			DC/TBD^§^		
**Genes associated with the phenotype**	Gene	OMIM number	Proportion attributed to pathogenic variants in each gene	Gene	OMIM number	Proportion attributed to pathogenic variants in each gene
	1. *FANCA*	607139	64%	1. *ACD* ^Ÿ^	609377	<1%
	*2. FANCB*	300515	2%	*2. CTC1*	*613129*	1–3%
	*3. FANCC*	613899	10%	*3. DCLRE1B/SNM1B/Apollo*	609683	<1%
	*4. FANCD1/BRCA2* ^œ,±^	600185	2%	4. *DKC1*/*NAP57* ^Ÿ^	300126	20–25%
	*5. FANCD2*	613984	3%	*5. NAF1*	617868	<1%
	*6. FANCE*	613976	1%	*6. NHP2*/*NOLA3* ^Ÿ^	606470	<1%
	*7. FANCF*	613897	2%	*7. NOP10*/*NOLA3*	606471	<1%
	*8. FANCG/XRCC9*	602956	8%	*8. PARN* ^Ÿ^	604212	<1%
	*9. FANCI*	611360	1%	*9. POT1*	606478	<1%
	*10. FANCJ/BRIP1* ^œ^	605882	2%	*10. RPA1*	179835	<1%
	*11. FANCL*	608111	<1%	11. *RTEL1* ^Ÿ^	608833	2–8%
	*12. FANCM*	609644	<1%	*12. STN1*/*OBFC1*	613128	<1%
	*13. FANCN/PALB2* ^œ,±^	610355	2%	13. *TERC*/*hTRz*	602322	5–10%
	*14. FANCO/RAD51C* ^œ^	602774	<1%	*14. TERT* ^Ÿ^	187270	1–7%
	*15. FANCP/SLX4*	613278	2%	15. *TINF2* ^£^	604319	12–20%
	*16. FANCQ/ERCC4/XPF*	133520	<1%	16. *WRAP53/TCAB1* ^Ÿ^	612661	<1%
	*17. FANCR/RAD51*	179617	<1%	*17. ZCCHC8*	616381	<1%
	*18. FANCS/BRCA1* ^œ^	113705	<1%	*Unknown*		20%
	*19. FANCT/UBE2T*	610538	<1%			
	*20. FANCU/XRCC2*	600375	<1%			
	*21. FANCV/MAD2L2/REV7*	604094	<1%			
	*22. FANCW/RFWD3*	614151	<1%			
**Inheritance patterns**	Autosomal recessive: 20 out of 22 *FANC* genes Autosomal dominant: *FANCR/RAD51* X-linked: *FANCB*			Autosomal dominant: DCLRE1B/SNM1B/Apollo, NAF1, RPA1, TERC/hTR, TINF2, and ZCCHC8 Autosomal dominant or autosomal recessive: ACD, PARN, RTEL1, and TERT Autosomal recessive: CTC1, NHP2/NOLA3, NOP10/NOLA2, POT1, STN1/OBFC1, WRAP53/TCAB1 X-linked: DKC1/NAP57		
**Pathophysiology**	DNA repair			Telomere maintenance		
**Diagnostic strategies**	Chromosome breakage analysis (gold standard) using DEB or MMC Genotyping of *FANC* genes			Telomere length (gold standard): southern blot, flow-FISH, or qPCR Genotyping of genes associated with DC/TBD phenotype		

^œ^Well-established cancer predisposition genes when they are in heterozygosis. ^±^ Genotypes associated with embryonal tumors (Wilms tumor, neuroblastoma, and brain tumors [most often medulloblastomas]) and acute myeloid leukemia at an early age. ^§^Some other genes have been associated with telomere maintenance but there is insufficient evidence to consider them as DC/TBD genes. ^£^Pathogenic variants in this gen are associated with Revesz syndrome. ^Ÿ^Pathogenic variants in this gen are associated with Hoyeraal-Hreidarsson syndrome.

DC/TBD, dyskeratosis congenita/telomere biology disorders; DEB, diepoxybutane; MMC, mitomycin C; OMIM, Online Mendelian Inheritance in Man.

(3, 7, 118, 123–130).

Genetic heterogeneity is also remarkable in FA. PVs in at least 22 genes that work together in the FA/BRCA pathway have been identified in patients with FA (131) (**Table 2**). The majority of genes have an autosomal recessive inheritance pattern, except for *FANCB*, which is X-linked and *FANCR/RAD51*, which is autosomal dominant (6, 132) (**Table 2**).

The physical phenotype in patients with FA is variable and multisystemic as depicted in **Figure 6** (37, 133). Classical abnormalities include those described in the VACTERL-H (Vertebral, Anal, Cardiac, Tracheo-esophageal fistula, Esophageal atresia, Renal, upper Limb, and Hydrocephalus) association (134, 135). From 5% to 30% of patients with FA meet the criteria for VACTERL-H (≥ 3 out of 8 features) (37, 134, 135). However, the clinician should suspect FA even if the patient has less than two VACTERL-H anomalies, especially if radial ray and kidney malformations coexist (136). Other common abnormalities are grouped in the PHENOS acronym (skin Pigmentation, small Head, small Eyes, Nervous system, Otology, and Short stature) (135). Overall, the most frequent anomalies are as follows: short stature, upper limb structural abnormalities, pigmentary skin changes, renal malformations, and microcephaly (37). Both acronyms can trigger an early suspicion of the disease, even before the onset of the hematooncological phenotype (136).

### 3.1 Neurological findings

Retrospective studies have shown that nervous system abnormalities are frequently found in both FA and DC/TBD (37, 117, 133, 137, 138). Structural neurological anomalies were reported in 40% of patients with FA, whereas 10–50% of patients with DC/TBD had these findings (37, 117, 133, 137, 138).

#### 3.1.1 Structural abnormalities

Case reports of patients with FA have described a wide range of neurological anomalies such as microcephaly, cerebral/cerebellum hypoplasia, small or abnormal pituitary gland, abnormalities of the corpus callosum, malformations of posterior fossa, hydrocephalus, and malignancies (36, 139–143). The identification of central nervous system (CNS) abnormalities increases when brain imaging is used. Brain magnetic resonance imaging is important for the thorough description of patients with FA, since almost 60% of patients have abnormal imaging (144, 145). When comparing brain images of patients with FA and age and sex-matched control population without intracranial pathology, it was found that 61% had at least one CNS or skull-based abnormality (144, 145). The most frequent structural CNS anomalies are localized in the midline, such as pituitary hypoplasia (46%), and in posterior fossa consisting of cysts, vermis, and ponto-cerbellar hypoplasia (32%) (144, 145). However, when the imaging studies were performed in patients with either neurological or endocrine manifestations, CNS abnormalities were found in 90% (146). In this group, the most frequent structural anomalies were posterior fossa abnormalities that required neurosurgery (146).

Genotype–phenotype association has been evaluated in patients with FA regarding CNS anomalies, microcephaly, and hydrocephalus. Structural CNS anomalies have been associated with PVs in ID2 complex genes, microcephaly with PVs in *FANCD1*, *FANCD2*, *FANCJ*, ID2 complex, downstream genes, and hydrocephalus with *FANCB* genotype (37).

The frequency of structural abnormalities in DC/TBD as discovered by systematic evaluation through brain magnetic resonance imaging is 57% (147). The brain structure that is predominantly affected is the cerebellum; the more frequent structural abnormality is cerebellum hypoplasia or atrophy, seen in 39% of patients with DC/TBD. Other frequent findings are cerebral atrophy (18%) and midline alterations such as corpus callosum abnormalities (16%) and small pons (11%) (147). The number of neuroimaging abnormalities has been found to be correlated with shorter telomeres, XL and AR inheritance patterns, and severe BMF (147). The same analysis for patients with DC/TBD showed much higher frequencies than in general population of *corpus callosum* anomalies, prominent *cisterna magna*, white matter lesions, and cavum *septum pellucidum*, and *cavum vergae* variants (147).

#### 3.1.2 Functional abnormalities

The information concerning functional neurological abnormalities in FA is scarce; a systematic approach to describe the functional neurologic phenotype in FA is missing. The available information comes from transversal case series analysis in which 32.5% of functional neurological involvement has been estimated. The identified abnormalities include intellectual disability, epilepsy, attention deficit hyperactivity disorder, and migraine (145, 146).

Any functional neurological disorder in DC/TBD has been described in around 50% of patients (147). Neuromotor findings include abnormal gait, dysarthria, incoordination, and ataxia (147). These types of alterations have been associated with shorter telomeres, HHS or RS, XL or AR inheritance patterns, and severe BMF (147). Some psychiatric manifestations have also been found in patients with DC/TBD (depression, anxiety or panic attacks, autism, and bipolar disorders) (147).

Most persons with DC/TBD have no significant developmental delay or intellectual disability, but the severe phenotypes HHS and RS do. HHS neurological phenotype is associated with cerebellum hypoplasia, microcephaly, and neurodevelopmental disorder (119–121), whereas RS includes bilateral exudative retinopathy, intracranial calcifications, and neurodevelopmental disorder (3, 122).

### 3.2 Ocular findings

Retrospective analyses have described that 11–20% of patients with FA have ophthalmological abnormalities (37, 133). Published cases have reported small eyes (148–150), strabismus (151, 152), epicanthal folds (153), ptosis, cataracts (154, 155), glaucoma (154, 156, 157), myopia (152), astigmatism, and retinopathy (154, 155, 158–160). Ocular alterations are also frequently described in DC/TBD (133), but their nature is different from what is seen in FA.

#### 3.2.1 Structural abnormalities

Intentional review of ocular characteristics identifies at least one abnormally small dimension of the eye structure in 95% of patients with FA (151). Cross-sectional studies with systematic evaluation have found microcornea in 46–100%, microphthalmia in 69–100%, small palpebral fissures in 75–94%, ptosis in 28–88%, hypotelorism in 25–43%, cataracts in 14–30%, limbal neovascularization in 26–28%, epicanthal folds in 0–9%, strabismus in 7.5%, and posterior embryotoxon in 5% of assessed patients (151, 161, 162). Compared with age- and gender-matched normal values, patients with FA have abnormally diminished: interpupillary distance, inner canthal distance, outer canthal distance, palpebral fissure length, palpebral fissure width, and corneal diameter (151, 161, 162). The interpupillary distance, outer canthal distance, and corneal diameter are smaller compared with other IBMFS (151). Specifically, the palpebral fissure length was significantly smaller than in DC/TBD (151). This is an interesting finding, since the short palpebral fissure is considered a part of the eye involvement in the PHENOS acronym (135) and seems to be specific of FA. The limbal neovascularization and cataract were more common in patients who underwent hematopoietic cell transplantation (HCT) (161). An association between small eyes and a *FANCD2* genotype has been found by a single publication (37).

Optic nerve hypoplasia, characterized by a decreased number of optic nerve axons (18), can manifest as small disc and cup areas in patients with FA (161, 162). The identification of nerve hypoplasia during eye fundus examination should prompt neuroimaging studies, since this ophthalmologic feature may be associated with midline cerebral structural defects, such as the absence of the *septum pellucidum*, agenesis of the *corpus callosum*, cerebral hemisphere abnormalities, or pituitary gland anomalies (163).

Around 30% of patients with DC/TBD have eye anomalies according to retrospective studies (116). Ocular size is not a main feature, only small outer canthal distance and palpebral fissure length have been reported (151). The most common findings in DC/TBD are nasolacrimal duct obstruction in 29% (164–166), retinopathy in 21%, trichiasis (165, 167), and entropion in 7% (151, 167). The spectrum of ocular anomalies also includes conjunctivitis (167), ectropion (168), corneal scarring (167), cataracts (169, 170), and retinal vasculopathy (171–174).

#### 3.2.2 Functional abnormalities

Regarding visual functions, 88% of evaluated patients with FA have abnormalities including motion problems (162). No systematic evaluation of functional alterations in DC/TBD have been performed.

Considering the high frequency of ocular abnormalities both in FA and DC/TBD, integral ophthalmic assessment at the time of diagnosis is important to procure patients with the benefit of prompt and specific interventions. Due to their frequencies, small ocular parameters should bring to mind an FA diagnosis and nasolacrimal duct obstruction a DC/TBD one.

An important consideration is that, besides ocular manifestations that are part of the primary diagnosis, patients with FA and DC/TBD are subject to receive treatments that affect the eye. In particular, steroids and HCT regimens are associated with an increased risk of glaucoma, cataract, corneal ulcers, cytomegalovirus retinitis, fungal endophthalmitis, and retinopathy (175, 176). A higher prevalence of these findings has been found in patients after HCT (162). Regular ophthalmological follow-up is important after HCT for early intervention to avoid complications.

### 3.3 Otological findings

The prevalence of otological problems in patients with FA varies from 10% to 20% according to literature reviews (1, 2). Hearing impairments are more frequent in FA compared with other IBMFS (177).

#### 3.3.1 Structural abnormalities

Structural ear anomalies of any kind have been found in more than half of patients after an intentional evaluation (177). As it appears to be the rule in FA, the structural abnormality range is wide. The following alterations have been described: small tympanic membrane in 62%, malformed malleus in 33-57%, bony islands at tympanic membrane in 48%, pinna malformations (including microtia) in 10-35%, abnormal course or prominence of the chorda tympani nerve in 34%, narrow external auditory canal in 32%, narrow internal auditory canal in 6%, and atresia of the external auditory canal in 3% (177, 178).

In sharp contrast, in DC/TBD structural, ear abnormalities have not been reported despite the intentional study of otologic features has been done (177).

#### 3.3.2 Hearing impairment

Hearing loss has been found in 36–55% patients with FA, with a high proportion of bilateral and asymmetric impairment (3–6). The most common type of hearing loss in FA is the conductive type, followed by sensorineural and, last, the mixed pattern (3–5). This translates into mild and moderate degrees of hearing impairment (177–180), particularly decreased hearing has been associated with the presence of structural ear anomalies (3). Assessment through the speech perception in noise test showed that up to 53% of evaluated patients had hearing impairment (178).

An analysis that sought associations of hearing loss with physical anomalies in FA found that 100% of patients with radial bone malformation had some hearing impairment; this association was statistically significant compared with patients without radius anomaly (177), which led to speculate that a developmental relationship between the radius and ear could exist (3). This is why otological findings, particularly associated with radial ray malformations and/or skin pigmentation changes, should prompt an evaluation for FA.

Genotype–phenotype association has been established between structural or functional otological anomalies and *FANCB* and *FANCD2* genotypes (37, 181).

Although hearing loss has not been commonly described in DC/TBD (117, 133, 138), intentional otological evaluation identified that 6% of patients have hearing problems, which is double than in healthy population through epidemiological studies (177, 182). In the general population, a link between telomere attrition and age-related hearing loss has been demonstrated (183). However, another cross-sectional study showed that telomere length was not associated with hearing acuity in healthy children or their midlife parents (184).

The genetic instability associated with FA and the shorter telomere length of DC/TBD have been associated with premature aging, which hypothetically might lead to progressive sensorineural hearing loss (177).

Structural or functional otological findings, or a combination of both, are common in patients with FA, and less frequent but seen as part of the DC/TBD spectrum. Formal hearing assessment, including otolaryngology evaluation, is recommended as part of the management of patients with FA and DC/TBD (177). The identification of otological abnormalities may lead to prompt intervention to reduce language complications through adequate rehabilitation interventions.

### 3.4 Oral findings

The oral cavity can be easily disregarded during physical examination, yet it can frequently hold key features to integrate a diagnosis. In patients with DC/TBD, some sort of dental alteration was found in 23% (185). On their part, almost half of patients with FA have been reported to have at least one mucosal lesion (186), and alterations in the number and shape of teeth have also been found (187, 188). Moreover, both for FA and DC/TBD, the extreme risk of cancer in this location requires diligent oral inspection (189).

#### 3.4.1 Dental organs

##### 3.4.1.1 Developmental abnormalities

Although developmental abnormalities in dental organs have been reported in both DC/TBD and FA, the spectrums are different. In DC/TBD, the reported anatomic differences are very prevalent as a decreased root/crown ratio has been identified in up to 75% of patients and mild taurodontia in 60% (190). On the other hand, the dental phenotype in FA has a wider spectrum, even though it is less constant: Microdontia has been reported in 44% (187), variations in teeth numbers have been found with dental agenesia identified in over 25% (187, 188), and supernumerary teeth in less than 10% (187).

##### 3.4.1.2 Decay, periodontal disease, and gingivitis

Common oral alterations such as caries and periodontal disease have also been found in patients with DC/TBD and FA (190); it is hard to control the multiple factors associated with these disorders in order to conclusively define if they belong in the phenotype of these uncommon syndromes. Nevertheless, these features have been described in both of these IBMFS. According to the DC registry, 17% of patients have extensive dental caries or loss of dental pieces (117); this has also been found in 35% of patients with FA (187). Gingivitis appears to be an even more prevalent feature in FA; it has been identified in nearly 50% (188, 191). Dental hygiene and access to stomatologic care are undoubtedly fundamental factors in the development of these type of oral manifestations.

#### 3.4.2 Lesions of the oral mucosa

Lesions of the oral mucosa are frequent in both of these syndromes: Transient blisters and erosion are early manifestations of DC/TBD (192), and mucosal lesions have been found in up to 42% of patients with FA (186). A recent extensive analysis of 279 patients with FA revealed that they have frequent synchronic and metachronic oral lesions (193).

##### 3.4.2.1 Leukoplakia

The WHO’s working expert group for potentially malignant oral disorders has conserved the leukoplakia definition as “*a predominantly white plaque of questionable risk having excluded (other) known disorders or disorders that carry no increased risk for cancer*” (194). This is a cardinal diagnostic feature in DC/TBD that is part of the classical clinical triad used for diagnosis suspicion; it has been reported in 78% of patients from the DC registry (117) and can be as frequent as 96% in certain literature reviews (185). The location of leukoplakia in DC/TBD patients is mainly found in the dorsal tongue followed by buccal mucosa (190). It has been hypothesized that the leukoplakia seen in patients with DC/TBD are hyperkeratotic scars that result from genetically mediated epithelial atrophy, pigmentation, and scarring (192). Leukoplakia is not exclusive of DC/TBD; after an intentional review of oral manifestations in FA, some authors have gone to hypothesize that this is an underreported feature as it was found in 12% of patients, located predominantly in the palate (186). Meanwhile, in the general population, leukoplakia has been found in less than 5% with a predominant location in the commissure or the buccal mucosa (195).

##### 3.4.2.2 Erythroid lesions

The second most frequent oral lesion in both DC/TBD and FA is erythroplakia (190, 193). In DC/TBD, the erythematous patches are more frequently found in the occlusal plane (190). These lesions, by their own, do not seem to be related to malignancy, but in the largest analysis of oral lesions in patients with FA, it was found that lesions that combined erythroplakia with erosion were highly correlated with a positive brush biopsy for malignancy (193) and are the ones that should be more closely followed up.

### 3.5 Gastrointestinal findings

The spectrum of gastrointestinal (GI) system alterations found in patients with FA an DC/TBD does not overlap, but they can equally impact the patient’s quality of life. In FA, GI alterations are mainly malformations that will usually require surgery (37, 133); meanwhile, in patients with DC/TBD, GI features are not congenital but appear later in life.

#### 3.5.1 Liver abnormalities

The GI organs can be affected in multiple ways in patients with DC/TBD. One of the largest cohorts of patients with DC/TBD described hepatic manifestations in 40% of patients (196). The range of hepatic involvement includes mild findings, such as hepatomegaly, inflammation, hemosiderosis, and fibrosis, to more severe manifestations such as cirrhosis and portal hypertension (115, 197, 198). Imaging evaluation has showed an increase in hepatic echogenicity in 39%, hepatomegaly in 26%, and nodular contour suggestive of cirrhosis in 25% of patients (196). Severe symptoms are more common in children and have been found in 16% of patients with DC/TBD. A study of 69 individuals from five unrelated families showed intrafamily variability in which penetrance had an important role and phenotype variability with a broad spectrum of liver disorders that included both inflammatory and fibrotic findings (197). Liver involvement and/or isolated liver fibrosis can also be a sole manifestation in patients with DC/TBD, a presentation more commonly associated with PVs in *TERT* rather than *TERC* or *TINF2* (196, 199). Although the specific pathophysiology has not been identified, some reports associate liver abnormalities with vascular anomalies (200).

Liver alterations in patients with FA are usually secondary to androgen therapy for BMF; they consist of hepatomegaly, increase in hepatic echogenicity, biliary duct dilation, and elevated liver enzymes (201). However, liver alterations have been described in 5/44 patients with FA in the absence of androgen therapy or another risk factors. The liver alterations found were very severe chronic cytolysis pattern, significant biological liver abnormalities, minor cytolysis pattern, hepatomegaly, and adenomas (201).

#### 3.5.2 Digestive tract abnormalities

The tubular part of the GI can also be affected in DC/TBD; esophageal stenosis has been identified in 10% of the patients; other alterations such as Schatzki’s ring, atrophic gastritis, esophageal intraepithelial lymphocytosis, inflammatory changes, and mild fibrosis of gastric *lamina propria* have been reported in lower frequencies (202). Although small and large bowels are not commonly affected, there are reports of pancolitis, epithelial sloughing, and increased apoptosis. Upper tract GI hemorrhage is an unusual manifestation that can be severe and deadly if not treated promptly. A multicenter review reported 16 patients with DC/TBD who presented with recurrent GI hemorrhage unresponsive to conventional management (203). Although no specific cause for the GI hemorrhage was identified, a higher risk for it has been established for patients with the presence of telangiectatic superficial vessels in the stomach and small intestine (203). PVs in the gene *TINF2* were more frequently detected in patients who had GI hemorrhage (203). Patients with PVs in this gene were overrepresented among patients who had pulmonary arteriovenous malformations (PAVMs) (204), which could suggest that a similar vascular lesion might be found in their GI tracts. Other nonspecific GI symptoms reported in patients with DC/TBD include early satiety, nausea, abdominal pain, solid dysphagia, failure to thrive, and watery or bloody diarrhea. Besides, DC/TBD, very short telomere length has been evaluated as a biomarker for hepatic involvement. Around 70% of the subjects with hepatic involvement have shorter telomeres (196).

In FA, anal and esophageal malformations, the GI anomalies included in the VACTERL-H association have a fairly low frequency of 5% (133, 205). According to Alter and Rosenberg’s literature review, of those patients with FA who have more than three VACTERL-H features, the Anal, Renal, Limb (ARL) combination is the third most frequent, and, overall, 34% of patients with FA and VACTERL-H have a GI malformation. The combination of both anal and esophageal malformations in a single patient with FA and VACTERL-H is infrequent as only 3% will have both (206). Other GI malformations reported in FA are atresia of jejunum, annular pancreas, exocrine pancreatic insufficiency, and intestinal malrotation (207). GI problems as a single feature of FA is rare. There are two case reports of patients diagnosed with FA after the recognition of esophageal atresia type III in the neonatal period (208). Regarding genotype–phenotype association, PVs in *FANCB* have been associated with anal anomalies, tracheo-esophageal fistula, and esophageal/duodenal atresia whereas *FANCN* only with anal malformations (37).

GI symptoms in patients with FA or TDC/BD with or without history of HCT or androgen therapy deserve evaluation. This could prevent severe complications such as GI bleeding, cancer, or liver dysfunction. An integral GI evaluation that includes radiological and/or biochemical studies is required in patients with DC/TBD in the interest of diagnosing any of the described liver anomalies (196, 209).

### 3.6 Cardiopulmonary findings

Both FA and DC/TBD have cardiopulmonary manifestations, yet each condition has preferent trophism for an organ. The heart is frequently affected in FA, and the lungs are usually spared whereas, in DC/TBD, the opposite is seen. Moreover, the type and pathophysiologic mechanisms leading to these features are particular to each condition.

#### 3.6.1 Cardiac abnormalities

The frequency of cardiovascular manifestations in patients with FA differs according to the type of study that reports it; it ranges from 13% according to a sound literature review to up to 55% if a systematic heart evaluation is performed (37, 206). The discrepancy on the reported data could be attributed to the fact that some cardiovascular anomalies are asymptomatic and consequently underdiagnosed. Cardiac structural abnormalities are frequently the third feature for a VACTERL-H diagnosis in patients with FA (37). The CRL (cardiac, renal, and limb) combination, with or without other features, was found in 54% of patients with FA (59/108) (37). There is a wide spectrum of structural heart defects reported in FA, mostly diagnosed at birth (37, 206). The most common cardiac defects documented are septal defects, but others such patent ductus arteriosus, coarctation, situs inversus, truncus arteriosus, bicuspid aorta, and a left persistent superior vena cava have also been reported (207, 210). An association between cardiac defects and a *FANCB* and *FANCI* genotypes has been found by a single publication (37).

In sharp contrast, only 1–4% of patients with DC/TBD are born with a congenital heart defect; the spectrum essentially consists of ventricular and atrial septal defects and dilated cardiomyopathy (103, 198). Congenital heart defects are one of the most common malformations in the population, presenting in around 1% of the population; it is not clear if DC/TBD have an intrinsic increased risk for congenital heart anomalies or if those who present them have a concurring disease of multifactorial etiology (211, 212).

#### 3.6.2 Pulmonary abnormalities

Pulmonary fibrosis is the best known complication in patients with DC/TBD, with a prevalence of 20% and is usually diagnosed beginning at the fourth decade of life (138, 213, 214). The characteristic radiological findings are diffuse interstitial markings (215). A cohort of 72 patients with idiopathic pulmonary fibrosis showed that this anomaly can occur as the first manifestation in patients with short telomeres and even in the absence of hematological findings (216). In a systematic review of patients with DC/TBD and pulmonary fibrosis, the early onset of this alteration (before 40 years of age) was associated with more severe and earlier onset of BMF (214). The same analysis found an association between PVs in *TINF2* gene and an early onset pulmonary fibrosis and BMF (214). Other pulmonary anomalies associated with DC/TBD are typical and atypical interstitial pneumonia, bronchiectasis, cysts, bronchiolitis obliterans, chronic hypersensitivity pneumonitis, and emphysema, which have been reported with a lower frequency in two small cohorts of patients with DC/TBD (217, 218).

Pulmonary disease is the second most common cause of death after BMF in patients with DC/TBD (213). Yet, in patients with PVs in genes with an AD pattern of inheritance (38.7%), pulmonary complications are the main cause of mortality (198). PAVMs have been reported in 3% of patients with classic DC (204, 219). A study of 13 unrelated patients with DC/TBD showed a median age of 15 years old for PAVMs diagnosis; 46% (6/13) of them had germline PVs in *TINF2* (204). The observed and reported frequencies are low, but this could result from failure to recognize PAVMs in virtue of shared symptoms with pulmonary fibrosis (103, 204). Further investigation is needed to establish the etiology of PAVMs in order to determine whether they are a consequence of telomere dysfunction or if they are associated with HCT, since they are a common finding in patients who undergo this procedure (198, 204). It is interesting that the phenotype of patients with DC/TBD due to PVs in *TINF2* has important vascular features such as PAVMs, GI hemorrhage, and in the exudative retinopathy found in RS.

In FA pulmonary abnormalities, either malformations or auto-immune process have been reported as coincidental findings; these are not fibrotic changes like the ones seen in DC/TBD. There are reports of two patients with FA who had a pulmonary arterio-venous fistula and single reports of pulmonary alveolar proteinosis, pulmonary glial heterotopia, and interstitial lung disease (220–223). Nevertheless, further investigation is needed to understand whether these pulmonary manifestations could be the direct consequence or FA and/or treatment for its complications.

Upon diagnosis, a complete cardiovascular examination in patients with FA is warranted due to the high incidence of structural heart defects; no further follow-up is needed if malformations are not detected. Meanwhile, pulmonary findings in patients with DC/TBD are only expected in symptomatic patients who will need follow-up with pulmonary function test and/or computed tomography scan to monitor disease progression.

### 3.7 Genitourinary findings

#### 3.7.1 Kidney and urinary tract abnormalities

The location of urinary anomalies is different between patients with DC/TBD and FA. Patients with FA usually have malformations of the upper urinary tract (224, 225), whereas fibrotic alterations of the lower urinary tract are found in patients with DC/TBD.

Alterations in the urinary system are seen in 22–50% of patients with FA (37, 207, 224, 226, 227). The combination of renal plus limb abnormalities (RL) can be seen in up to 20% of patients with FA, in those who happen to also have a VACTERL-H diagnosis, the frequency goes up to 94% (1, 6). This highlights the importance of renal evaluation when FA is suspected. The spectrum of urinary anomalies is heterogenous; it includes renal hypoplasia or aplasia, dysplastic or ectopic kidneys, crossed fused ectopia of kidneys, double ureters, cysts, and horseshoe kidney (224, 228). These renal anomalies can frequently be accompanied by urinary tract infections, which are key indicators of a renal malformation particularly if present frequently and in early childhood (224). Hydronephrosis of unknown etiology has been reported in three cases with FA (36, 229, 230).

In a comprehensive literature review that included 380 probands, patients with null genotypes, irrespective of the gene and those with PVs in *FANCB* and *FANCJ*, had a positive association with renal malformations (37).

Urinary tract anomalies in patients with DC/TBD most commonly manifest in the lower tract and are diagnosed later in life once associated symptoms develop (117). Urethral stricture or stenosis is reported in at least 5% of patients, but it can increase up to 10% in men (198). Bleeding or acute urinary retention syndrome is a sign consistent with the suspicion of a urethral stenosis (231). Although most urinary anomalies in patients with DC/TBD are age dependent, there are case reports of patients with malformations such as renal agenesis (232), horseshoe kidney, bilateral renal cysts, and duplication of renal collecting system and ureters (198). Renal structural abnormalities are common congenital malformations in the general population that occur in one of 1,000 births; this apparently isolated renal malformations reports might be coincidental findings (21).

#### 3.7.2 Genitalia abnormalities

Genital abnormalities are not as common in patients with FA. Men present genital anomalies in 12–16% (37, 227) with a lower frequency in women, but this could be due to an underdiagnosis since the internal nature of the female tract requires imaging or specialized maneuvers for a thorough evaluation (37). Male patients can have poor genital development with micro-penis, hypospadias, and cryptorchidism or even major malformations such as complete transposition of penis and scrotum (207, 226). Uterine malformations such as bicornate uterus and labial fusion have also been reported (207, 233).

Reported genitalia manifestations in DC/TBD are phimosis, hypospadias, penile leukoplakia as well as undescended testes and in fewer cases hypogonadism (103, 198, 234). Women have also presented vaginal atrophy and leukoplakia (103).

Although the genitourinary system can be affected on both patients with FA and DC/TBD, the major difference is in the part of the genitourinary system that is affected. The upper urinary tract corresponds to FA, and the lower urinary tract corresponds to DC/TBD. In addition, the time of diagnosis differs, because the anomalies associated with DC/TBD tend to be progressive and identified later in life, whereas those associated with FA are congenital malformations and could be detectable from birth.

### 3.8 Dermatological findings

Dermatologic findings are the cornerstone of the classic DC triad. Reticulate hyperpigmentation and nail dysplasia are individually present in nearly 90% of patients with a DC/TBD (116). The dermatological phenotype may not be present at the time of diagnosis but, as many DC/TBD features, insidiously develops or becomes more pronounced over time. The skin pigmentation usually appears between the ages of 5 and 10 years, and nail dystrophia can appear in the first months of life, although is usually present toward adolescence (235). Skin pigmentation changes are most commonly found in sun-exposed areas (neck, trunk, and both extremities); they consist of reticulate hyperpigmentation, which can be associated with hypopigmentation, atrophy, poikiloderma, and telangiectasias (116–118). Nails appear dystrophic with longitudinal ridging and are usually more severely affected in the hands than the feet (236, 237). Other cutaneous manifestations include hair involvement, such as premature hair graying or loss and even alopecia, palmoplantar hyperkeratosis, and eczema (213, 235–242).

Skin findings in FA are so frequent that they have a place in the PHENOS acronym. Retrospective studies have found skin anomalies in around 40% of patients (37, 133). The most common alterations are pigmentary changes such as café-au-lait macules, generalized hyperpigmentation, and hypopigmented macules (37, 133). Cross-sectional studies that intentionally looked for skin changes in patients with FA have found at least one pigmentary anomaly in 82–97% of evaluated patients (243–245). The most frequent finding was café-au-lait macules, with a mean of 7.5 per patient; they were distributed across the whole body (243). Hypopigmented macules, seen in 46% of patients, were the second most common anomaly (243). Recently, freckle-like macules were noted in 24.7%; they were characteristically located at flexing areas that included lateral neck, popliteal fossa, and axillary, inguinal, and suprapubic creases (243). Other less common findings were confluent flexural hyperpigmentation and diffuse macules (243). The combination of hypopigmented along with hyperpigmented macules was the most frequently identified at 45.2% (243, 244). No associations were found between skin findings and radiation exposure, cutaneous graft-versus-host disease, or HCT. However, increasing age was associated with an increase in the odds of freckle-like macules (243). Sweet syndrome has been recurrently described in patients with FA (246–249). This neutrophilic dermatosis characterized by painful violaceous plaques and nodules with dermal neutrophilic infiltrate can occur as a paraneoplastic syndrome (250); therefore, in patients with FA, it should alert of the possible development of an oncological process. According to a comprehensive literature review, a higher proportion of patients with PVs in *FANCD1* and *FANCD2* had at least one skin anomaly compared with all other genes (37).

Patients with FA or DC/TBD who undergo an HCT can have long-term skin findings (251); however, these anomalies are different from those due to the disease.

Awareness of the high frequency of dermatological abnormalities presentation in patients with FA or DC/TBD is essential to increase diagnostic suspicion and favor early diagnoses.

### 3.9 Skeletal findings

These anomalies represent an area of sharp contrast between FA and DC/TBD. Skeletal abnormalities are found in over half of patients with FA, whereas these alterations are only incidentally described in DC/TBD (3, 118).

#### 3.9.1 Upper limb abnormalities

The characteristic upper limb malformations linked to FA are radial ray anomalies (radius, thumb, and/or thenar alterations), which are part of the VACTERL-H acronym (135, 206, 252–255). Classically, FA has been associated with severe and, sometimes, bilateral upper limbs anomalies (253), which is why a FA diagnosis may be overlooked in patients with mild anomalies, such a thenar hypoplasia.

Around 50% of cases with FA have some kind of hand or arm anomalies (133, 256), but 40% are described as radial ray anomalies (37). Retrospective analyses have found the following radial ray malformations: thumb hypoplasia in 32%, preaxial polydactyly (including triphalangeal thumb) in 19%, thumb aplasia in 12%, absent or hypoplastic radii in 8–16%, and hypoplastic thenar eminence in 3% (256). All cases with an abnormality of the radius had a concomitant thumb anomaly (37, 257, 258), which is contrasting with thrombocytopenia and absent radii syndrome where thumb not severely affected (139). Upper limb alterations were more frequently mild and bilateral than those severe and unilateral (253, 257, 258). Actually, bilateral hand anomalies had a 4.6 odds ratio for positive DEB test result when contrasted to unilateral involvement (258). Other type of hand anomalies including clinodactyly, brachymesophalanges, and brachydactyly have been reported (37, 256).

The relevance of radial ray abnormalities as a pivot sign for suspicion of FA is indisputable; children with a limb anomaly were diagnosed two years younger compared with those without, and the severity of the thumb hypoplasia was inversely associated with the age at diagnosis (257). When a patient with radial ray alteration is being evaluated, clinicians should consider that the more severe the radial ray defect is, the more possible a syndromic condition is (253, 259); also, it must be kept in mind that patients with FA may have very discreet upper limb alterations that can be missed by an inexperienced appraisal.

A study determined that incidence of FA among children with congenital thumb anomalies was 7% (258). The same analysis showed that patients diagnosed with FA represent 1% of the patients with any type of congenital thumb anomalies and 3% of those with thumb hypoplasia (258). Most patients tested for FA had other physical abnormalities, and all of those who had a positive FA test result had café-au-lait macules (258).

#### 3.9.2 Vertebrae and other skeletal abnormalities

The spectrum of these type of anomalies includes hemivertebrae, hypoplastic vertebrae, vertebral fusion (Klippel-Feil syndrome), spina bifida, coccygeal aplasia, scoliosis, kyphosis, and atlantoaxial instability (37, 133, 260). A recent prospective study published that vertebrae anomalies are found in almost 50% of patients after intentional evaluation (245), which is in sharp contrast with the 4% described in the literature (37, 133). Some reports have depicted less frequent anomalies, such as rib hypoplasia, Sprengel deformity, and hip dysplasia (149, 225, 261–264).

In patients with FA and VACTERL-H, 27% had the VL combination that includes vertebral and upper limb alterations (206). Orthopedic surgeons should be attentive, as they may come across patients with FA in their everyday practice. They are in an advantageous position to identify patients with FA before the onset of hematologic manifestations, which is essential for an appropriate treatment and surveillance.

Upper limb malformations were more frequent in patients with PVs in *FANCB* and *FANCD2* (37). *FANCD2* genotype was also associated with lower limb anomalies, specifically hip involvement, whereas *FANCB* was associated with vertebral defects (37, 149).

### 3.10 Growth and endocrine findings

The endocrine system has not been studied in patients with DC/TBD; although there are some anecdotal cases in the literature, hormone deficiencies seem uncommon in these patients (3). In contrast, almost 80% of patients with FA have been shown to have an endocrine abnormality; this striking frequency of endocrine affection has been proposed to be the consequence of affected secretory cells due to DNA damage caused by excessive reactive oxygen species (265). HCT, a fundamental therapy for BMF both in patients with FA and DC/TBD can contribute in endocrine conditions such as diabetes, dyslipidemia, hypogonadism, thyroid, adrenal, and pituitary dysfunction (266).

#### 3.10.1 Short stature, intrauterine growth retardation, and microcephaly

Comprehensive knowledge of the clinical presentation of DC/TBD comes from analysis of features from patients who participate in the DC registry (117). Data from 118 male patients with a clinical diagnosis of DC/TBD showed that short stature is part of the phenotype in almost 20% of patients, whereas intrauterine growth retardation and microcephaly was documented in just over 7% and 6%, respectively (117). Short stature does not appear to be due to growth hormone deficiency, since there is a single report of a child with growth hormone deficiency in whom brain magnetic resonance imaging was normal (267). Analysis of other cohorts has confirmed the frequency of short stature but found lower frequencies of IUGR and microcephaly (185). Precisely, these two features are found in over 96% of patients attained by the severe variant of DC/TBD known as HHS (268).

Meanwhile, growth deficiency is such an important piece of the FA phenotype that two features have been integrated into the PHENOS acronym (P: skin Pigmentation; H: small Head; E: small Eyes, N: Nervous system, O: Otology, S: Short stature), which recapitulates some of the physical manifestations of FA (37). The data from different cohorts have documented short stature in 46–60% of patients with FA (265, 269, 270), whereas microcephaly has been documented in 33% (270). This phenotype has been further delineated to specify that about half of patients are small for gestational age and that only 25% of them will reach normal stature (271). Moreover, in FA, concomitant endocrinopathies are frequent. The combination of growth hormone deficiency (reported in 12% of patients with FA) and hypothyroidism will result in shorter stature in these subjects (265).

According to a genotype–phenotype association analysis, short stature was more frequent in patients with null PVs and those with PVs in downstream genes, whereas microcephaly was associated with null genotypes irrespective of the gene and patients with PVs in *FANCD1*, *FANCD2*, *FANCJ*, ID2 complex, and downstream genes (37).

#### 3.10.2 Other endocrine abnormalities

Thyroid function is frequently compromised in patients with FA; hypothyroidism was found in up to 61% of children and 37% of adults (265). Primary hypothyroidism is predominant, although some patients have central hypothyroidism secondary to hypothalamic dysfunction (271). Meanwhile, in DC/TBD, hypothyroidism has only been reported in two isolated cases (272, 273).

Glucose metabolism has also been found to be disturbed in patients with FA: Impaired fasting glucose or glucose intolerance was documented in 24% of patients whereas diabetes mellitus in 10% (269) and insulin resistance in over 40% (269, 270). Meanwhile, in DC/TBD, such metabolic alterations do not appear to be frequent (274). This finding is intriguing, because several cross-sectional studies have shown that patients with type 2 diabetes mellitus had telomeres significantly shorter than control subjects (275–277). These studies have also found a significant inverse relationship between oxidative damage and telomere length (275–277). In mice with short telomeres, the β-cell function, insulin secretion, and glucose metabolism are impaired recapitulating the early stages of human type 2 diabetes mellitus (278). Nevertheless, to our knowledge, this has not been systematically evaluated in patients with DC/TBD.

### 3.11 Fertility and pregnancy findings

There are fertility issues in both DC/TBD and FA, although, like endocrine abnormalities, they are more frequent in patients with FA. According to the DC registry, hypogonadism is found in approximately 6% of patients (117). Meanwhile, this is a more pervasive feature in FA where both men and women are similarly affected: Hypogenitalism characterized by small testes and phallus has been reported in 64% of male patients with FA, and over 75% of female patients have premature ovarian failure (271).

Ovarian reserve of patients with FA has been intentionally explored by measuring anti-Mullerian hormone (AMH) (279). Ovarian compromise appears to be a component of the FA phenotype; it is present from prepuberal stages and is not a complication derived from HCT, since both transplanted and non-transplanted patients show low AMH levels (279, 280). This finding has also been replicated in DC/TBD patients (median AMH level 0.55 ng/ml), but AMH levels were not as low as what was found in patients with FA (median 0.05 ng/ml) (14, 15). These low AMH levels are in accordance with expected fertility issues.

Pregnancies in patients with FA are rare; less than 50 pregnancies, regardless of transplant status, have been reported in the literature (4). Reproductive complications in women with FA include decreased fertility, preeclampsia, increased premature births and C-sections, and declined maternal hematopoiesis during pregnancy (281). Reproductive and pregnancy outcomes in women with DC/TBD have recently been explored in a group of 26 women older than 16 years who had a total of 80 pregnancies (282). Reproductive complications in this group were similar to those reported in patients with FA, with decreased fertility as 23% of patients required some sort of fertility treatment, a high rate of fertility loss and recurrent pregnancy loss, as well as more frequent preeclampsia, preterm births and a higher C-section delivery rate. Moreover, hematological abnormalities during pregnancy or delivery occurred in a quarter of pregnancies (282).

### 3.12 Immunologic findings

FA and DC/TBD share the common defining feature of BMF. In most patients with the classical presentation of either condition, the impairment of the white blood cell lineage translates as subtle immunologic abnormalities. However, there are seldom cases, particularly those belonging to the HHS subtype of DC/TBD, for whom severe immune manifestations are the rule.

There are a few studies that describe the immune phenotype of patients with FA. In a large cohort of patients with FA with advanced BMF, immunologic data showed hypogammaglobulinemia, reduced absolute lymphocyte counts, and increased serum TGF-β and Il-6 (283). The National Cancer Institute (NCI) IBMFS cohort looked at the data from patients belonging to the whole spectrum of severity of the BMF; this well-controlled study corroborated that the immunologic phenotype of patients with FA consisted of decreased immunoglobulins and absolute lymphocyte counts as well as detailed that B and NK deficiencies occur at a younger age whereas T-cell deficiencies later in life (284). Moreover, by meticulously comparing the data from patients and their unaffected relatives, the study revealed that abnormal immune functions in FA are more prevalent in adults than in children (284). Although cytokine dysregulation with over production of TNF-α and IFN-γ have been proposed to contribute to an altered hematopoiesis in patients with FA (285, 286), these finding were not corroborated in the NCI cohort (284).

Regarding DC/TBD, the clinical presentation of the immune phenotype is wide. It is a cornerstone of HHS, in which severe combined immunodeficiency attains all patients (287). In the NCI cohort study of immunological features of patients with DC/TBD, the absolute lymphocyte counts and their subset numbers were reduced in children and in the lower range for adults; IgA tended to be increased in adults but no other alteration of immunoglobulins was documented (284). In contrast to what was seen in FA, the immune deficiency was more important in children than in adults; this difference may be due to the fact that one-third of DC/TBD patients in this study had an HHS phenotype, which presents in pediatric age (284). Although severe combined immunodeficiency is a feature of HHS, not all patients with DC/TBD will have such a severe immune outcome; common variable immunodeficiency has also been reported as the initial presentation in DC/TBD (288).

### 3.13 Oncological findings

#### 3.13.1 FA and DC/TBD as cancer predisposition syndromes

**Figure 7:**
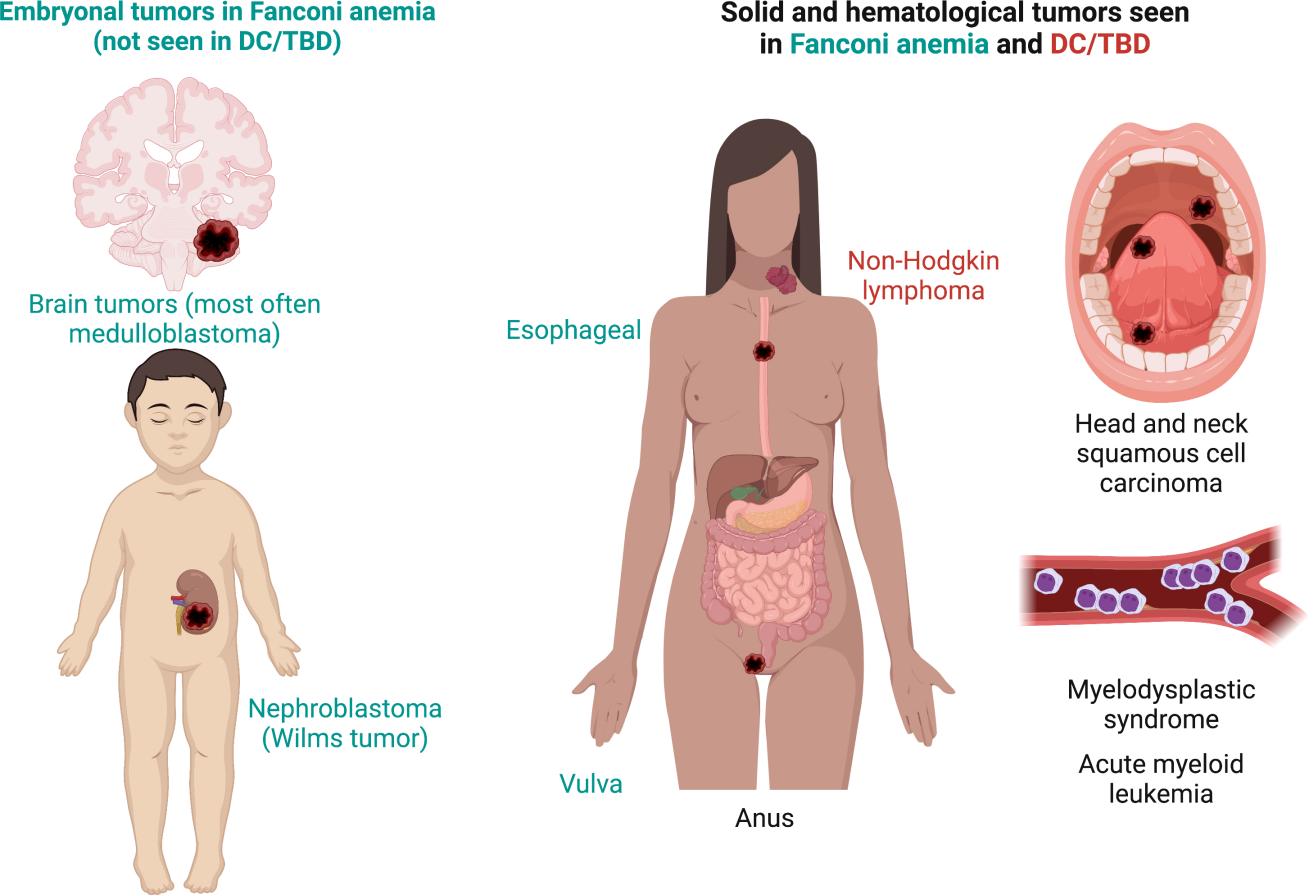
Fanconi anemia and DC/TBD share a common spectrum of cancer. SCCs may be found in gums, tongue, palate, or any other mucosal surface in the mouth; SCCs can also be found elsewhere in the gastrointestinal tract particularly in the anus and for patients with FA in the esophagus. The hematologic cancer phenotype consisting of myelodysplastic syndrome and acute myeloid leukemia is shared by both disorders. In patients with DC/TBD, non-Hodgkin lymphoma is added to the oncological spectrum. Gynecologic SCC is a distinctive feature of FA. Also, a subgroup of patients with FA with *FANCD1/BRCA2* and *FANCN/PALB2* genotypes have been associated with the development of embryonal tumors (Wilms tumor, neuroblastoma, and brain tumors [most often medulloblastomas]) and acute myeloid leukemia at an early age. Features exclusive to FA are in green, those exclusive to DC/TBD are in red, and those shared by both disorders are in black. DC/TBD, dyskeratosis congenita/telomere biology disorders; FA, Fanconi anemia; SCCs, squamous cell carcinomas.

Cancer predisposition genes are defined as those genes where germline PVs and likely pathogenic variants (LPVs) confer high or moderate risks of cancer (greater than twofold relative risks) (289). FA and DC/TBD are known to be cancer predisposition syndromes. The cumulative incidence of cancer is close to 20% by age 65 years in both diseases (189, 219, 290). An important consequence of the hematological management improvement is that cancer has become a frequent cause of death in patients with FA and DC/TBD. The shared and contrasting oncological phenotype for both diseases is summarized in **Figure 7**.

#### 3.13.2 Cancer in FA

Patients with FA have a much greater risk of developing specific types of cancer than general population. FA confers a 700-fold higher risk of developing acute myeloid leukemia (AML) and head and neck squamous cell carcinoma (HNSCC) compared with non‐FA population (291, 292). Moreover, the median age of diagnosis of these neoplasms is significantly younger than that of sporadic cancer (293). Patients with FA are also highly susceptible to squamous cell cancer (SCC) in other locations, such as cervix, anus, and vulva (294). Patients with FA have a 3,000-fold higher risk of vulvar SCC than the general population. In addition, SCCs in FA are usually more aggressive and often diagnosed in more advanced stages of the disease (290).

Patients with biallelic PVs in *FANCD1/BRCA2* and *FANCN*/*PALB2* have very high risks of malignancies, and they can develop embryonal tumors which are not seen in patients with FA who have PVs in other *FANC* genes. The embryonal cancers reported in these patients are as follows: Wilms tumor, neuroblastoma, and brain tumors (most often medulloblastomas) (262). A recent analysis of 71 patients with PVs in *FANCD1/BRCA2* and 16 patients with *FANCN*/*PALB2* genotype confirmed this association (295). This publication also showed that patients with these genotypes can have multiple cancers and an earlier age of the tumor presentation (295).

Physicians treating patients with cancer should be alert of the *FANCD1*/*BRCA2* and *FANCN*/*PALB2* associated malignancies and consider the possibility of FA diagnosis. Wilms tumor, neuroblastoma, and brain cancer could appear before the hematological manifestations. Importantly, the combination of these embryonal tumors, or the association with AML, is a strong indicator for FA diagnosis. Patients with biallelic PVs in *FANCD1*/*BRCA2* or *FANCN*/*PALB2* should have cancer surveillance at very young age, particularly focused on the associated cancers.

##### 3.13.2.1 Heterozygous PVs in FA and the development of cancer

A major concern is whether the risk of cancer is increased in heterozygous individuals. Five *FANC* genes are well-established cancer predisposition genes when they are in heterozygosis. These genes are as follows: *FANCD1/BRCA2*, *FANCJ/BRIP1*, *FANCN/PALB2*, *FANCO/RAD51C*, and *FANCS/BRCA1* (7).

It is unclear whether heterozygous PVs of the other *FANC* genes can confer cancer predisposition. McReynolds et al., and Berwick et al., found that the heterozygous of PVs in other *FANC* genes do not have an increased risk of cancer (296, 297). However, some studies suggest that the *FANC* genes could increase the risk of developing cancer in heterozygous individuals. In **Table 3**, we summarized the evidence related to risk of cancer for each *FANC* gene (7, 293, 297–311).

**Table 3:** Heterozygous Pathogenic Variants in FANC Genes and Cancer Development.

*FANC* gene	Cancer risk in heterozygotes	Risk of breast cancer	Risk of ovarian cancer	Other types of cancer	References
*FANCA*	Possible	Insufficient data Strength of evidence: limited	Unknown	Unknown	(298, 299)
*FANCB*	Unknown	Unknown	Unknown	Unknown	
*FANCC*	Possible	Insufficient data Strength of evidence: limited	Unknown	Insufficient data: early-onset pancreatic cancer Strength of evidence: limited	(7, 297)
*FANCD1/BRCA2*	Yes	Absolute risk: >60% Strength of evidence: Very strong (with predisposition to ER+ disease)	Absolute risk: 13–29% Strength of evidence: Very strong	Pancreatic cancer Absolute risk: 5–10% Strength of evidence: Very strong Prostate cancer and melanoma Absolute risk: Unknown or insufficient evidence	(300)
*FANCD2*	Possible	Unknown	Unknown	Insufficient data: T cell acute lymphoblastic leukemia, testicular seminoma, HNSCC Strength of evidence: limited	(293, 301)
*FANCE*	Possible	Unknown	Unknown	Insufficient data: HNSCC Strength of evidence: limited	(293)
*FANCF*	Possible	Unknown	Unknown	Insufficient data: HNSCC Strength of evidence: limited	(302)
*FANCG/XRCC9*	Possible	Unknown	Unknown	Insufficient data: early-onset pancreatic cancer, HNSCC Strength of evidence: limited	(7, 302, 303)
*FANCI*	Possible	Unknown	Unknown	Insufficient data: uterine serous papillary carcinoma, well-differentiated SCC of tongue Strength of evidence: limited	(304)
*FANCJ/BRIP1*	Yes	Absolute risk: Insufficient data to define Strength of evidence: Limited; potential increase in female breast cancer (including triple negative)	Variant is estimated to be 5.8–12% though lifetime risk of developing ovarian cancer	Unknown	(305, 306)
*FANCL*	Possible	Unknown	Unknown	Insufficient data: HNSCC Strength of evidence: limited	(293)
*FANCM*	Possible	Absolute risk: Insufficient data to define Strength of evidence: strong (with predisposition to triple-negative and early-onset breast cancer)	Unknown	Unknown	(307, 308)
*FANCN/PALB2*	Yes	Absolute risk: 41–60% Strength of evidence: Strong (with overrepresentation of triple-negative disease	Absolute risk: 3–5% Strength of evidence: Strong	Pancreatic cancer Absolute risk: 5–10% Strength of evidence: Limited Other cancers Unknown or insufficient evidence	(309)
*FANCO/RAD51C*	Yes	Absolute risk: 15–40% Strength of evidence: Strong for ER/PR-negative breast cancer	Unknown	Absolute risk: >10% Strength of evidence: Strong	(310)
*FANCP/SLX4*	Unknown	Unknown	Unknown	Unknown	
*FANCQ/ERCC4/XPF*	Unknown	Unknown	Unknown	Unknown	
*FANCR/RAD51*	Unknown	Absolute risk: 15–40% Strength of evidence: Strong for ER/PR-negative breast cancer	Unknown	Absolute risk: >10% Strength of evidence: Strong	
*FANCS/BRCA1*	Yes	Absolute risk: >60% Strength of evidence: Very strong (with predisposition to triple-negative disease)	Absolute risk: 39–58% Strength of evidence: Very strong	Pancreatic cancer Absolute risk: ≤5% Strength of evidence: Strong Prostate cancer: Insufficient evidence	(300)
*FANCT/UBE2T*	Possible	Insufficient data Strength of evidence: limited	Unknown	Unknown	(311)
*FANCU/XRCC2*	Possible	Insufficient data Strength of evidence: limited	Unknown	Unknown	(304)
*FANCV/MAD2L2/REV7*	Unknown	Unknown	Unknown	Unknown	
*FANCW/RFWD3*	Unknown	Unknown	Unknown	Unknown	

The strength of evidence categories is based on the NCCN guidelines. Very strong: Prospective cohort studies in a population-based setting have demonstrated risk. Strong: Traditional case–control studies or more than three case–control studies including those with cases ascertained by commercial laboratories or those without controls from the same population. Traditional case–control study: A retrospective study that compares patients with a disease or specific outcome (cases) with patients without the disease or outcome (controls). Limited: Small sample size or case series. None.

ERs, estrogen receptors; HNSCC, head and neck squamous cell carcinoma; NCCN, National Comprehensive Cancer Network; PR, progesterone receptor; SCC, squamous cell carcinoma.

The detection of PVs in these cancer predisposition genes is important, since genetic counseling and several cancer surveillance measures can be offered to carriers. Chemoprevention and surgeries such as double mastectomy and risk-reduction salpingo-oophorectomy are options (123). Patients with PVs in *FANCD1/BRCA2*, *FANCS/BRCA1*, and *FANCN/PALB2* are candidates to treatment with poly(ADP-ribose) polymerase (PARP) inhibitors. One of the more recent studies is the Olympya trial, where patients with HER2-negative early breast cancer and germline PVs or LPVs in *FANCD1/BRCA2* and *FANCS/BRCA1* are treated with adjuvant Olaparib (312). Treated group had a significantly longer survival free of invasive or distant disease than placebo (312). PARP inhibitors were demonstrated to be effective in cancers with HR deficiency phenotype beyond PVs in *FANCD1/BRCA2* or *FANCS/BRCA1* (313).

#### 3.13.3 Cancer in DC

The most frequent malignancies present in patients with DC/TBD are HNSCC, AML, non-Hodgkin lymphoma, and anal SCC (219).

The first study that quantified the risk of cancer in patients with DC/TBD was the NCI IBMFS prospective cohort. According to this study, after 15 years of follow-up, patients with DC/TBD had an 11-fold higher risk of developing cancer than the general population, specifically, a 195-fold higher risk of leukemia and a 1,154-fold higher risk of tongue cancer (189, 219). Other reported malignancies were esophageal, rectal, endometroid, and cervical cancers. It is difficult to establish a genotype–phenotype association in DC/TBD. However, some data associate *DKC1*, *TERC*, *TERT*, *TINF2*, and *WRAP53* genes with a higher risk of cancer (198). Patients who developed cancer from the DC/TBD NCI cohort presented different patterns of inheritance: 48.1% had AD-non*TINF2* disease, 29.6% had AR/XLR disease, 3.7% had *TINF2*-associated disease, and 18.7% had an unknown mode of inheritance (198). Regarding the type of cancer, 71.4% of the leukemias and 40% of the solid tumors were developed by patients with AD disease and 35% of the solid tumors were developed by patients with AR/XLR disease. Five patients (three *TINF2*-associated diseases, two AR/XLR) developed a solid malignancy after HCT (198).

There is a lack of information regarding the risk of cancer in heterozygous carriers of PVs in genes associated with DC/TBD phenotype. While PVs in the *TERT* are highly penetrant and rare, more frequent and low penetrant single nucleotide polymorphisms (SNPs) have been linked to TBD, although they are not considered cancer predisposition syndromes. Most SNP carriers in the general population remain disease free. However, SNPs in *TERT* have been associated with cancer (314). For example, four TERT heterozygous variants (p.A243V, p.T726M, p.A1062T, and p.V1090M) were found in 3.3% of patients diagnosed with hepatocellular carcinoma associated with cirrhosis (315, 316). Moreover, the p.A1062T variant reported a threefold higher allele frequency in patients with AML and has also been found in higher frequency in cirrhotic patients (316). The p.A279T variant reported a fivefold higher allele frequency in patients with esophageal carcinoma (317).

## 4 Conclusions

FA and DC/TBD are IBMFS with genetic instability and cancer predisposition. A similar hematooncological presentation must compel the clinician to consider these two diseases as differential diagnoses during evaluation. Specialized testing such as chromosomal breakage and telomere length may be a common practice in BMF clinics in many developed countries but, worldwide, this is not the norm. Despite technological advances and the decreasing cost of DNA sequencing, there are significative disparities in access to genomic medicine, and diagnosis through high-throughput methods is available only to a small fraction of the world’s population. This is why, in countries where access to specialized tests is limited if not impossible, clinical assessment is king. The one-to-one comparison presented in this review may be used as guidance to integrate a clinical suspicion. Both disorders affect multiple systems and have pleiotropic manifestations. By and large, intentional evaluation results in a higher detection rate of specific features, particularly those that do not give symptomatology but need special tests or maneuvers to uncover them. It is important to keep in mind that there are age-dependent features that might need to be reevaluated later in the life of these patients. Their hematological and oncological manifestations overlap importantly. However, despite both IBMFS having extramedullary features, a discerning analysis of their type and time frame of appearance can clinically distinguish the two disorders. This distinction may permit hand-tailored follow-up to intentionally identify and care for features specific to each diagnosis as well as better guide the confirming test to request. Moreover, a precise diagnosis can further impact on the family as a whole, as this may allow the discussion of theoretical recurrence risks.

The genetic pathway disrupted in each of these conditions, hints as to why, despite presenting features in the same organs, their type and spectrum are distinct. In patients with FA, the failure in DNA repair appears to influence the availability of stem cells during morphogenesis. Also, DNA repair is necessary to maintain the pool of pluripotent stem cells during embryogenesis (318), possibly affecting migrating patterns (224). The latter may explain why many of the features included in VACTERL-H and PHENOS acronyms belong to the malformation category. Since equivocal formation occurs during embryogenesis, physical abnormalities found in FA are usually present before birth. They might not be detected immediately unless they represent a critical danger to the patient’s life, such as severe cardiac malformations, esophageal atresia, or anal malformations.

In comparison, physical findings in DC/TBD have an insidious evolution and are related to a scarring phenotype that results from fibrosis. We propose that frequent anomalies seen in at least 20% of patients with DC/TBD could be grouped in the GINPOD acronym (**G**astro**I**ntestinal [including liver and esophageal], **N**eurological, **P**ulmonary, **O**cular, and **D**ermatological [including skin, nails, and leukoplakia]). Telomere stability is altered in DC/TBD; this has a particular impact in tissues that depend on continuous replacement like epithelia. The maintenance of an adequate number of cells is dependent in telomere length; therefore, telomere attrition severely limits the cell proliferation and tissue regeneration capacities, leading to a replacement of healthy tissue by fibrotic one (46). Telomere metabolism disruption can also lead to a population of cells with genomic damage progress to cancer.

The cytogenetic phenotype of patients with FA is constant throughout life and is present in all cellular types; these qualities have made the cytogenetic test the gold standard for FA diagnosis. On the contrary, in patients with DC/TBD, having short telomeres does not translate into a consistent cytogenetic phenotype that can be used for diagnosis. The characteristic DC/TBD telomeric shortening, present since birth and evidenced by measuring telomere length, can progress with age and may become evident later in life. This progression could be related with the anticipation seen in some families with DC/TBD (319, 320). In accordance with this, higher frequencies of chromosomal aberrations have been observed in older members than in younger members of the same family (116). Further analysis of the DC/TBD cytogenetic phenotype, using next generation sequencing, FISH-banding, and M-FISH, could help elucidate the chromosome consequences of the telomere attrition.

Last but not least, discovery of genes associated with the FA and DC/TBDs phenotypes remains active. There are a few known patients with a cytogenetic diagnosis of FA (positive chromosome breakage test) in whom the genotype causing it has not been identified yet despite genetic analysis of known FANC genes. This incomplete genotype issue is also true for patients with telomeres below the first percentile for age. PVs in genes long-time known to protect the genome's integrity, like dominant PVs in RPA1, have recently been recognized as a cause of DC/TBDs (321, 322). This issue is expected to be solved by more powerful and more readily available genotyping techniques.

## Author contributions

MF-R: Conceptualization, Data curation, Formal analysis, Supervision, Writing: Review and Editing. BG: Conceptualization, Data curation, Formal analysis, Supervision, Writing: Review and Editing. PL-A: Data curation, Formal analysis, Writing: Review and Editing. Rt’H: Data curation, Formal analysis, Writing: Review and Editing. TW-O: Data curation, Formal analysis, Supervision, Writing: Review and Editing. SF: Conceptualization, Data curation, Formal analysis, Supervision, Fundingacquisition, Writing: Review and Editing. AR: Conceptualization, Data curation, Formal analysis, Supervision, Funding acquisition, Writing: Review and Editing. All authors contributed to the article and approved the submitted version.

## Funding

AR received support from Dirección General de Asuntos del Personal Académico, Universidad Nacional Autónoma de México (PAPIITprojectIA205022) and Consejo Nacional de Ciencia y Tecnología (CONACyT project 319344). SF received support from Dirección General de Asuntos del Personal Académico, Universidad Nacional Autónoma de México (PAPIIT project 205120), Consejo Nacional de Ciencia y Tecnología (SEPCONACyT project 243102), and Recursos Fiscales Instituto Nacional de Pediatría (project 2020/012).

## Acknowledgments

We acknowledge the Laboratorio de Citogenética team for the support and help. Special appreciation to Armando Hernández, Paulina Gómez-Moreno, and Rubí Espinosa-Curiel for their critical reading of this manuscript and their valuable comments.

## Conflict of interest

The authors declare that the research was conducted in the absence of any commercial or financial relationships that could be construed as a potential conflict of interest.

## Publisher’s note

All claims expressed in this article are solely those of the authors and do not necessarily represent those of their affiliated organizations, or those of the publisher, the editors and the reviewers. Any product that may be evaluated in this article, or claim that may be made by its manufacturer, is not guaranteed or endorsed by the publisher.
